# Novel 1-hydroxy phenothiazinium-based derivative protects against bacterial sepsis by inhibiting AAK1-mediated LPS internalization and caspase-11 signaling

**DOI:** 10.1038/s41419-022-05151-7

**Published:** 2022-08-18

**Authors:** Chuang Yuan, Kelong Ai, Menghua Xiang, Chengliang Xie, Mingyi Zhao, Ming Wu, Hongli Li, Yueren Wu, Yueqing Cao, Can Li, Yanjun Zhong, Xiaomeng Pei, Helen Ka Wai Law, Liqian Gao, Qicai Xiao, Xinyu Yang

**Affiliations:** 1 Department of Hematology, Xiangya Hospital, Central South University, Changsha, 410000 PR China; 2 Xiangya School of Pharmaceutical Sciences, Central South University, Changsha, 410000 PR China; 3 School of Pharmaceutical Sciences (Shenzhen), Sun Yat-sen University, and Shenzhen Campus of Sun Yat-sen University, Shenzhen, 518107 PR China; 4 Department of Hematology, The Seventh Affiliated Hospital, Sun Yat-sen University, Shenzhen, 518107 PR China; 5 ICU Center in the Second Xiangya Hospital, Central South University, Changsha, 410000 PR China; 6 Department of Health Technology and Informatics, The Hong Kong Polytechnic University, Hong Kong, PR China; 7 National Clinical Research Center for Geriatric Disorders, Xiangya Hospital, Central South University, Changsha, 410000 PR China

**Keywords:** Drug development, Bacterial infection

## Abstract

Sepsis is a life-threatening syndrome with disturbed host responses to severe infections, accounting for the majority of death in hospitalized patients. However, effective medicines are currently scant in clinics due to the poor understanding of the exact underlying mechanism. We previously found that blocking caspase-11 pathway (human orthologs caspase-4/5) is effective to rescue coagulation-induced organ dysfunction and lethality in sepsis models. Herein, we screened our existing chemical pools established in our lab using bacterial outer membrane vesicle (OMV)-challenged macrophages, and found 7-(diethylamino)-1-hydroxy-phenothiazin-3-ylidene-diethylazanium chloride (PHZ-OH), a novel phenothiazinium-based derivative, was capable of robustly dampening caspase-11-dependent pyroptosis. The in-vitro study both in physics and physiology showed that PHZ-OH targeted AP2-associated protein kinase 1 (AAK1) and thus prevented AAK1-mediated LPS internalization for caspase-11 activation. By using a series of gene-modified mice, our in-vivo study further demonstrated that administration of PHZ-OH significantly protected mice against sepsis-associated coagulation, multiple organ dysfunction, and death. Besides, PHZ-OH showed additional protection on *Nlrp3*^*−/−*^ and *Casp1*^−*/*−^ mice but not on *Casp11*^*−/−*^*, Casp1/11*^*−/−*^*, Msr1*^*−/−*^, and AAK1 inhibitor-treated mice. These results suggest the critical role of AAK1 on caspase-11 signaling and may provide a new avenue that targeting AAK1-mediated LPS internalization would be a promising therapeutic strategy for sepsis. In particular, PHZ-OH may serve as a favorable molecule and an attractive scaffold in future medicine development for efficient treatment of bacterial sepsis.

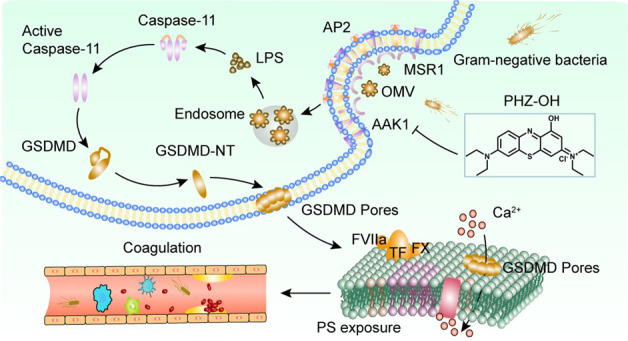

## Introduction

Sepsis, a syndrome with dysregulated host response to infection, is the leading cause of death worldwide [1, 2]. To date, clinical treatments of sepsis are mainly based on infection-prevention and life-supporting techniques, as efficient targeting medicines against sepsis are still scant due to the insufficient understanding of the explicit underlying mechanism. Therefore, development of novel therapeutic agents, especially those with favorable efficacy and explicit mechanism, are highly imperative in the treatment of sepsis.

Caspase-11 (human orthologs caspase-4 or caspase-5) is a pattern-recognition receptor (PRR) of innate immune system for the recognition of intracellular lipopolysaccharide (LPS)-the major pathogenic component of gram-negative bacteria [3–5]. We have previously demonstrated that caspase-11-mediated gasdermin D (GSDMD) pores magnify phosphatidylserine exposure of macrophages, thus enhancing the activity of tissue factor (TF) and subsequently promoting the coagulation cascades [6]. Coagulation is physiologically essential to trap invading microorganisms, thereby preventing microorganism diffusion and infection progression [7]. However, infection-induced sepsis often leads to excessive coagulation activation, which may result in life-threatening disseminated intravascular coagulation (DIC), and likely to induce multiple organ dysfunction and eventually death [2, 8, 9]. Our previous study showed that caspase-11 or GSDMD (the downstream) deficiency significantly attenuates LPS-induced DIC and subsequent death [6]. Also, we found that blocking type 1 IFNs pathway reduces hepatocyte-derived HMGB1, which dampens HMGB1-assistant LPS internalization and caspase-11-dependent DIC. [10]. Thus, inhibition of caspase-4/5/11 pathway would be a promising strategy to rescue coagulation syndrome and lethality in bacterial sepsis.

Clinical epidemiological studies have shown that gram-negative bacteria are the predominant pathogenic bacteria of sepsis-induced DIC [11]. Outer membrane vesicle (OMV), a compartment commonly secreted from gram-negative bacteria, is capable of delivering LPS into the cytosol of host cells, and thus results in caspase-11 activation [12]. In this study, we established a cell-based high-throughput drug screening system by using OMV-stimulated mice peritoneal macrophages [13, 14], and identified a novel phenothiazinium-based derivative, 7-(diethylamino)-1-hydroxy-phenothiazin-3-ylidene-diethylazanium chloride (PHZ-OH), which significantly suppressed OMV-mediated caspase-11 activation. Of note, administration of PHZ-OH attenuated coagulation activation, multiple organ injury and death in both lethal bacterial sepsis and sterile endotoxemia. The protective effects of PHZ-OH were mainly ascribed to blocking the AP2-associated protein kinase 1 (AAK1)-mediated endocytosis of LPS, thereby reducing the activation of caspase-11. Taken together, our findings suggest that pharmacologically targeting AAK1-mediated LPS internalization is a valuable therapeutic option for sepsis. PHZ-OH, a new and promising inhibitor of AAK1, may facilitate the future discovery of novel agents for efficient treatment of coagulation syndrome, organ dysfunction, and death in bacterial sepsis.

## Results

### 1-hydroxy phenothiazinium-based small molecule inhibits OMV-mediated caspase-11 signaling

**Fig. 1:**
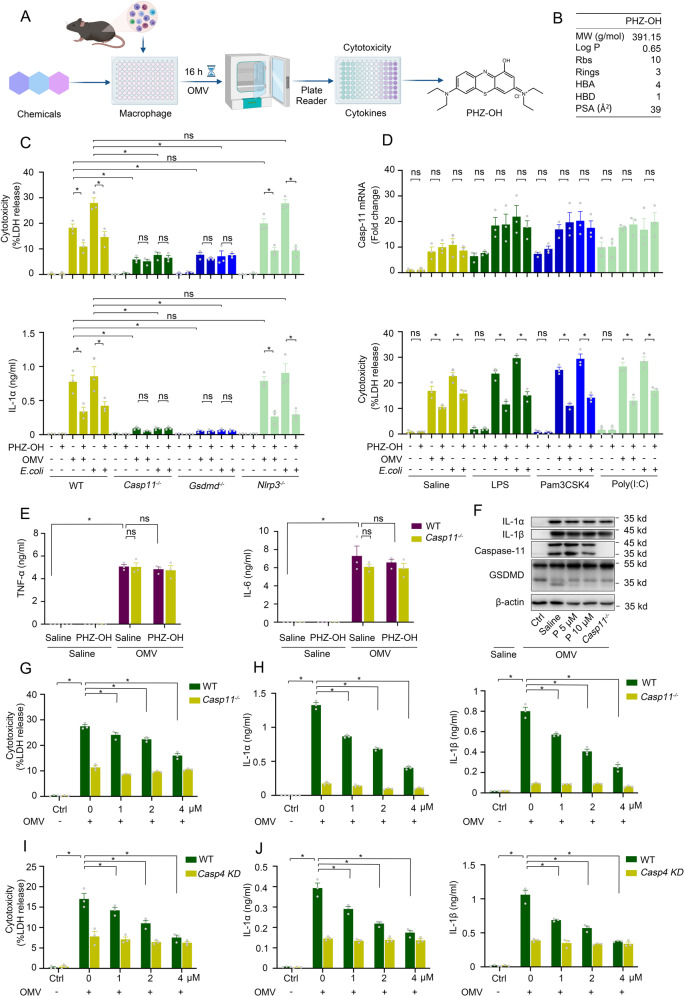
A 1-hydroxy phenothiazinium-based small molecule inhibits OMV-mediated cytotoxicity and cytokine release in vitro. **A** Scheme showing the screening strategy to identify potent inhibitors of caspase-11 signaling; **B** Drug-like properties of PHZ-OH; **C** Cytotoxicity and IL-1α secretion of wild type (WT), *Casp11*^*−/−*^, *Gsdmd*^*−/−*^, or *Nlrp3*^*−/−*^ mouse peritoneal macrophages under a treatment of PHZ-OH (5 μM), and/or a challenge of OMV (10 μg/mL) or *E. coli* (a MOI of 10) for 16 h; **D** Caspase-11 mRNA level and cytotoxicity of macrophages primed using LPS (1 μg/mL), Pam3cSK4 (1 μg/mL) or Poly(I:C) (1 μg/mL) for 3 h and challenged with OMV (10 μg/mL) or *E. coli* (a MOI of 10) for 16 h in the presence or absence of PHZ-OH (5 μM); **E** TLR4-dependent cytokines (TNFα and IL-6) released from WT or *Casp11*^*−/−*^ macrophages under a treatment of saline or PHZ-OH (5 μM), and/or a challenge of OMV (10 μg/mL) for 16 h; **F** Western blots for IL-1α, IL-1β and caspase-11 expression and GSDMD cleavage in saline-treated or OMV-challenged macrophages under a treatment of saline or different doses of PHZ-OH (P, 5 or 10 μM); **G**–**J** LDH (G or I) and IL-1α as well as IL-1β (**H** or **J**) released from mouse peritoneal macrophages (WT and *Casp11*^*−/−*^) or THP-1 cells (Transfected with control or caspase-4 siRNA), respectively, in the presence of different concentrations of PHZ-OH (1, 2, 4 μM) and challenged with OMV (10 μg/mL) for 16 h. Data are shown as mean ± SEM of three independent experiments. *P < 0.05. ns, not significant.

To identify novel and efficient inhibitors of caspase-11 signaling, we screened our existing chemical pools established in our lab by using mice peritoneal macrophages under a challenge of bacterial outer membrane vesicle (OMV)-the major transporter of LPS into the cytosol for caspase-11 activation [12] (Fig. 1A). We found that PHZ-OH, with excellent physiochemical and pharmaceutical properties according to Lipinski, Hann, and Veber’s rules [15–17] (Fig. 1B, Scheme S1, and Figs. S1–S4), displayed significant inhibition on OMV-induced pyroptosis. Given that caspase-11 activation not only induces GSDMD-associated cell death and the release of inflammasome-independent interleukin-1α (IL-1α), but also triggers NLRP3 inflammasome and the mature and secretion of inflammasome-dependent IL-1β and IL-18 [18], we thus challenged WT, *Casp11-, Gsdmd-* and *Nlrp3-*deficient primary peritoneal macrophages with OMV or *Escherichia coli* to verify the involvement of caspase-11 signaling. It was found that OMV or *E. coli*-induced cell death and IL-1α/β secretion were largely reduced by deleting caspase-11 and GSDMD, whilst deficiency of NLRP3 only blocked the inflammasome-dependent IL-1β release but not cell death and IL-1α secretion (Figs. 1C and S5A), indicating the caspase-11-dependent cell killing and cytokine release. Moreover, PHZ-OH significantly inhibited the OMV- or *E. coli-*induced cell death and IL-1α secretion in WT and *Nlrp3*-deficient but not *Casp11*- and *Gsdmd*-deficient macrophages, suggesting PHZ-OH is an inhibitor of caspase-11 signaling (Fig. 1C).

It has been recognized that the priming signal, commonly stimulated by toll-like receptors (TLRs), upregulates the expression of caspase-11 and thus facilitates caspase-11-dependent pyroptosis [3, 18]. To reveal if PHZ-OH exerts the inhibitory effect via inhibiting the priming signal, we treated macrophage with PHZ-OH after a priming of LPS (TLR4 agonist), Pam3cSK4 (TLR1/2 agonist) or Poly(I:C) (TLR3 agonist). It was found that priming of TLR agonists boosted the expression of caspase-11 but did not directly lead to cytotoxicity and IL-1 release until the challenge of OMV or *E. coli* (Figs. 1D and S5B). Meanwhile, treatment with PHZ-OH did not affect TLR-dependent caspase-11 expression but significantly attenuated OMV- or *E. coli*-induced cell death and IL-1α/β secretion (Figs. 1D and S5B). In addition, depletion of Myd88, the downstream of TLRs, almost completely blocked the release of TLR-related cytokines, tumor necrosis factor α (TNFα) and interleukin-6 (IL-6) (Fig. S5C), and reduced the inflammatory pyroptosis in OMV-challenged macrophages (Fig. S5D), while additional treatment with PHZ-OH further promoted the reduction of pyroptosis at the basis of Myd88 deficiency (Fig. S5D). Moreover, TNFα and IL-6 were not significantly altered by the treatment of PHZ-OH (Figs. S5C and 1E). Western blots also showed that PHZ-OH significantly inhibited caspase-11-dependent GSDMD cleavage rather than the expression of caspase-11 and IL-1α/β (Fig. 1F). These results suggest that PHZ-OH exerts the inhibition on caspase-11 signaling independent of TLRs. Besides, we found an apparent dose-dependent activity of PHZ-OH on WT rather than *Casp11*-deficient macrophages challenged with OMV (Fig. 1G, H). To further confirm the activity on human source samples, we treated THP-1 cells with PHZ-OH and OMV using knocking down caspase-4 (human ortholog of caspase-11) as the negative control [19]. The results showed that PHZ-OH demonstrated similar dose-dependent inhibition effects on WT but not caspase-4 knockdown macrophages in terms of death and cytokine releases (Fig. 1I, J). Taken together, these results show that PHZ-OH could prevent OMV-mediated pyroptosis by selectively inhibiting caspase-4/5/11 pathway in vitro.

### Administration of PHZ-OH alleviates organ dysfunction and lethality in septic mice

**Fig. 2:**
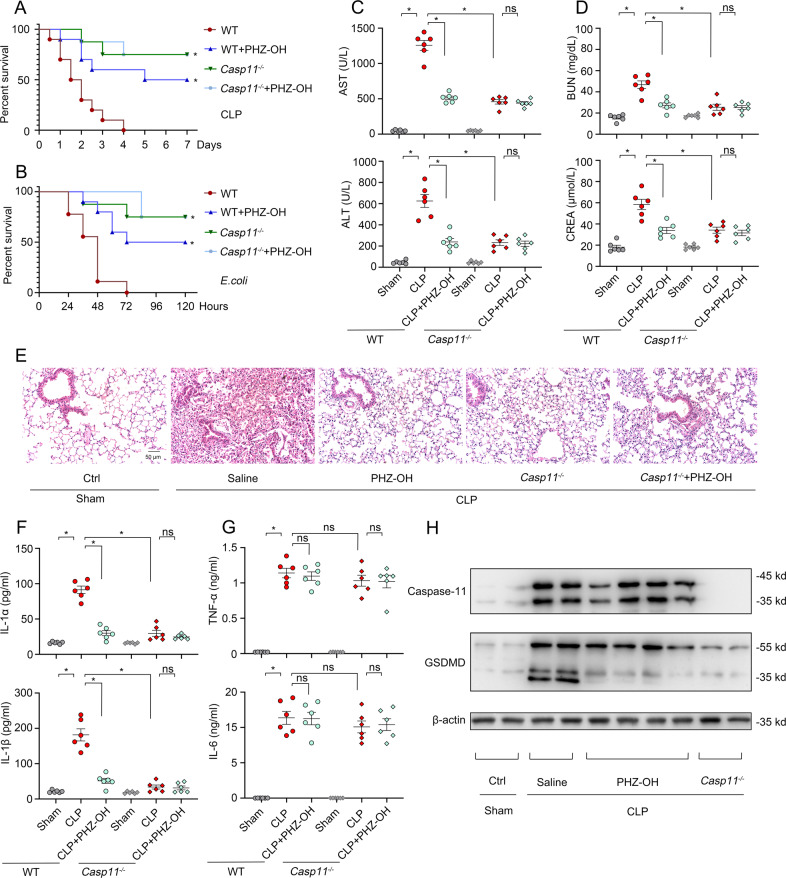
PHZ-OH attenuates organ dysfunction in bacterial sepsis via inhibiting caspase-11 signaling. Survival curves of WT and *Casp11*^*−/−*^ mice treated with saline or PHZ-OH (5 mg/kg) and subsequently challenged with cecal ligation and puncture (CLP, ligating 75% of the cecal) (**A**) or *E. coli* (ATCC 29522, 10^9^ CFU per mice) (**B**); **C**–**H** WT and *Casp11*^*−/−*^ mice were treated with saline or 5 mg/kg of PHZ-OH 30 min prior to a challenge of CLP (ligating 75% of the cecal) or not (six mice per group). The plasma, the lung and the liver were sequentially harvested 12 h after the challenge of CLP, and stored for further experiments. Variations of plasma-associated parameters including aspartate aminotransferase (AST) and alanine transaminase (ALT) (**C**), and blood urea nitrogen (BUN) and creatinine (CREA) (**D**); **E** Hematoxylin and Eosin (H&E) staining of the lung; Plasma levels of IL-1α and IL-1β (**F**), and TNFα and IL-6 (**G**); **H** Western blots showing caspase-11 expression and GSDMD cleavage in the liver (representative of six mice per group). Data are mean ± SEM of six mice in one experiment. *P < 0.05. ns, not significant. Scale bar = 50 μm.

To investigate the potential effects of PHZ-OH on sepsis, WT and *Casp11*^−/−^ mice were administrated with saline or PHZ-OH prior to a challenge of cecal ligation and puncture (CLP, polymicrobial sepsis) or *E. coli* (Gram-negative sepsis). Similar to the deficiency of caspase-11, administration of PHZ-OH significantly improved the survival rate in WT bacteria-septic mice, while without providing additional effects on *Casp11*^*−/−*^ mice, indicating PHZ-OH could specifically prevent caspase-11-dependent septic death (Fig. 2A, B). In view of the fact that interventions in clinics are commonly given after the onset of sepsis, we administrated PHZ-OH after the operation of CLP or the injection of *E. coli*. Gratifyingly, similar results were found and could be afforded on caspase-11-dependent protection in the treatment of bacterial sepsis (Fig. S6).

To investigate whether PHZ-OH attenuates organ dysfunction in sepsis, the plasma levels of alanine transaminase (ALT), aspartate aminotransferase (AST), blood urea nitrogen (BUN), and creatinine (CREA) were determined, by which the dysfunction levels of the liver and the kidney are demonstrated. We found that CLP-augmented ALT, AST, BUN and CREA were significantly inhibited by the administration of PHZ-OH (Fig. 2C, D). Similarly, genetically deleting caspase-11 attenuated the organ dysfunction, and no further improvements were observed under PHZ-OH treatment of *Casp11*^*−/−*^ mice (Fig. 2C, D). In addition, H&E staining confirmed that PHZ-OH significantly attenuated sepsis-induced lung injury, in line with the *Casp11*^*−/−*^ mice group (Fig. 2E). Moreover, inhibition effects on the plasma levels of caspase-11-related IL-1α and IL-1β, but not TLR4-related TNFα and IL-6, were observed in PHZ-OH-treated mice, which are essentially consistent with the results of the *Casp11*^*−/−*^ mice (Fig. 2F, G). As indicated by western blot, PHZ-OH also dampened caspase-11-dependent GSDMD cleavage but not TLR4-regulated expression of caspase-11 (Fig. 2H). Thus, these results demonstrate that PHZ-OH significantly prevents sepsis-induced organ dysfunction and lethality by selectively inhibiting caspase-11 signaling.

### Protective effects of PHZ-OH against sepsis do not rely on the inhibition of NLRP3 inflammasome

**Fig. 3:**
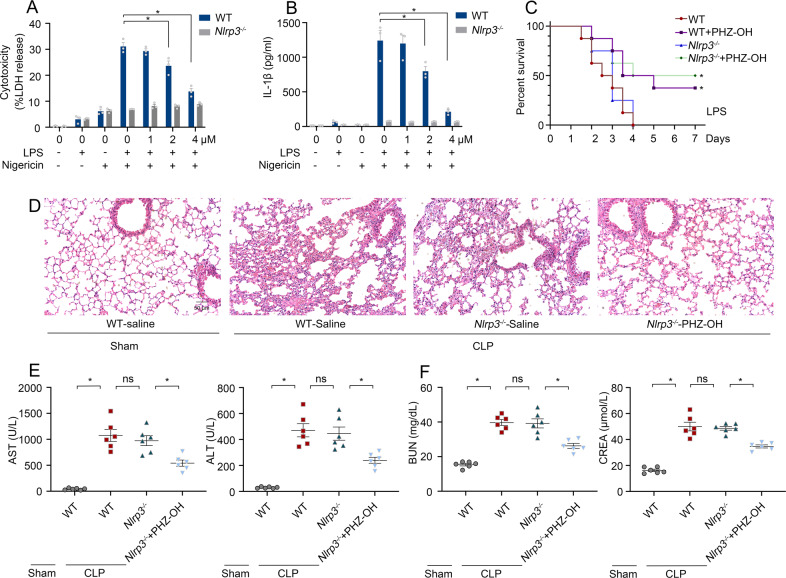
PHZ-OH protects against bacterial sepsis independent of NLRP3 inflammasome. Cytotoxicity (**A**) and IL-1β release (**B**) of mouse peritoneal macrophages primed using LPS (1 μg/mL) for 3 h and treated with nigericin (10 μM) for 1 h in the presence or absence of PHZ-OH (1, 2, 4 μM); (**C**) Survival rates of WT and *Nlrp3*^*−/−*^ mice that were treated with saline or PHZ-OH (5 mg/kg) prior to a challenge of CLP; Lung injury (**D**), liver (**E**) and kidney dysfunction (**F**) of WT and *Nlrp3*^*−/−*^ mice that were treated with saline or PHZ-OH (5 mg/kg) 30 min prior to a challenge of CLP or not (Samples were obtained 12 h after the challenge). Data are mean ± SEM of six mice in one experiment. *P < 0.05. ns, not significant. Scale bar = 50 μm.

As PHZ-OH inhibits inflammasome-dependent IL-1β secretion from OMV- or *E. coli*-challenged macrophages (Fig. S5A), its effects on NLRP3 inflammasome should be identified. We thus treated peritoneal macrophages of WT and *Nlrp3*-deficient mice with LPS plus nigericin (NLRP3 inflammasome inducer) in the presence or absence of PHZ-OH. Interestingly, PHZ-OH demonstrated dose-dependent inhibition on NLRP3-induced cell death and IL-1β release, and 4 μM of PHZ-OH almost completely blocked the canonical pyroptosis to a level as that of *Nlrp3*-deficient macrophages (Fig. 3A, B). To determine the effects of PHZ-OH and NLRP3 in in-vivo study, we subjected WT and *Nlrp3*^*−/−*^ mice to CLP after administration of PHZ-OH or not, and it was discovered that CLP-induced mice death was not significantly altered by deleting NLRP3 alone but improved by an additional administration of PHZ-OH (Fig. 3C). In addition, similar trends were found in organ injury and dysfunction, in which sepsis-induced lung injury (Fig. 3D) and impaired function of the liver and the kidney (Fig. 3E, F) were attenuated under the administration of PHZ-OH rather than by deleting NLRP3. These results were phenocopied in similar experiments using *Casp1*^*−/−*^ mice (Fig. S7). Taken together, these results suggest that PHZ-OH protects against organ dysfunction and lethality in sepsis independent of NLRP3 inflammasome.

### PHZ-OH protects against sepsis by inhibiting caspse-11 signaling in coordination of its antibacterial property

**Fig. 4:**
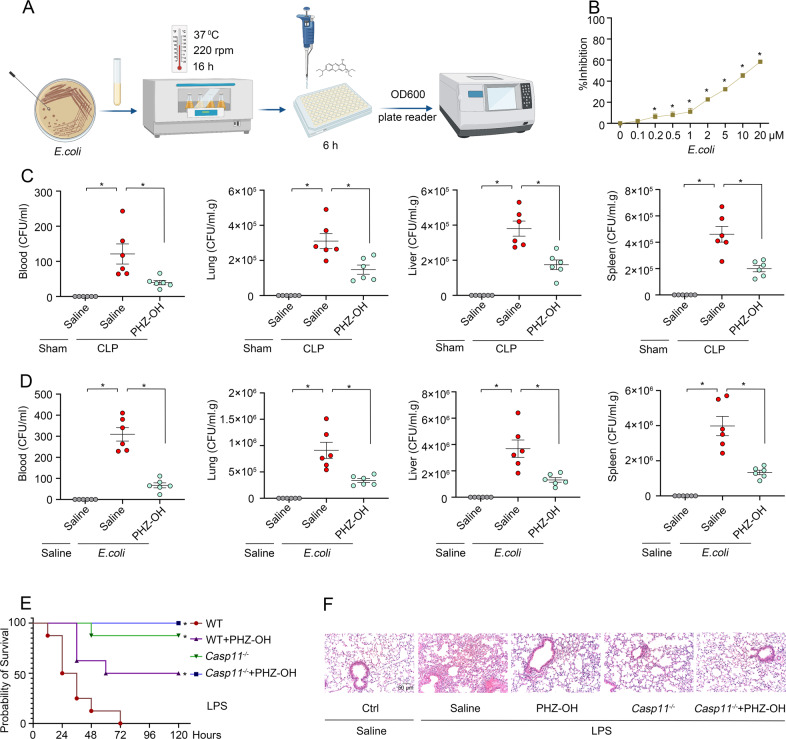
PHZ-OH attenuates bacterial sepsis in coordination of its antibacterial properties. **A** Schematic illustration of the process of antibacterial activity examination; **B** Antibacterial effects of PHZ-OH at various doses against *E. coli* (10^6^ CFU/ml for 6 h); Bacterial burdens of the plasma, the lung, the liver, and the spleen harvested 12 h after the challenge of CLP (**C**) or *E. coli* (**D**). Mice were treated with saline or 5 mg/kg of PHZ-OH 30 min prior to a challenge of CLP (ligating 75% of the cecal) or *E. coli* (ATCC 29522, 10^9^ CFU per mice); **E** Survival curves of WT and *Casp11*^*−/−*^ mice treated with saline or PHZ-OH (5 mg/kg) 30 min prior to a challenge of LPS (25 mg/kg); **F** Lung injury of WT and *Casp11*^*−/−*^ mice treated with saline or PHZ-OH (5 mg/kg) 30 min prior to a challenge of LPS (25 mg/kg) or not for 12 h. Data are mean ± SEM of six mice in one experiment. *P < 0.05. ns, not significant. Scale bar = 50 μm.

It is well-known that phenothiaziniums also possess outstanding antibacterial activities [20, 21], we thus examined the antibacterial effects of PHZ-OH against *E. coli* using standard activity assessment assay (Fig. 4A). The results showed that PHZ-OH demonstrated an excellent dose-dependent antibacterial effect (Fig. 4B). To identify whether the antibacterial property of PHZ-OH contributes to the protective effects towards bacterial sepsis, we determined the bacterial burden in the blood, the lung, the liver and the spleen of mice challenged with CLP or *E. coli*. To our delight, PHZ-OH dampened the bacterial burden in the circulation and parenchymal organs in both models, including the liver, the lung, and the spleen (Fig. 4C, D). To further separate the effects of the antibacterial and anti-caspase-11 properties of PHZ-OH in the prevention of sepsis, we next subjected mice to a sterile endotoxemia model. Consistent with our aforementioned results (Fig. 2A, B), caspase-11 deletion nearly completely blocked LPS-induced mice death (Fig. 4E) and attenuated lung injury (Fig. 4F), further implicating the pivotal role of caspase-11 on the pathogenesis of gram-negative sepsis. Moreover, administration of PHZ-OH significantly enhanced the survival rate of WT mice challenged with high dose of LPS (endotoxemia model), but it did not afford further improvement in *Casp11*^*−/−*^ mice. These results thus suggest that PHZ-OH prevents gram-negative sepsis by inhibiting caspase-11 pathway in coordination with its intrinsic antibacterial property.

### PHZ-OH protects mice via attenuating coagulation in gram-negative bacterial sepsis

**Fig. 5:**
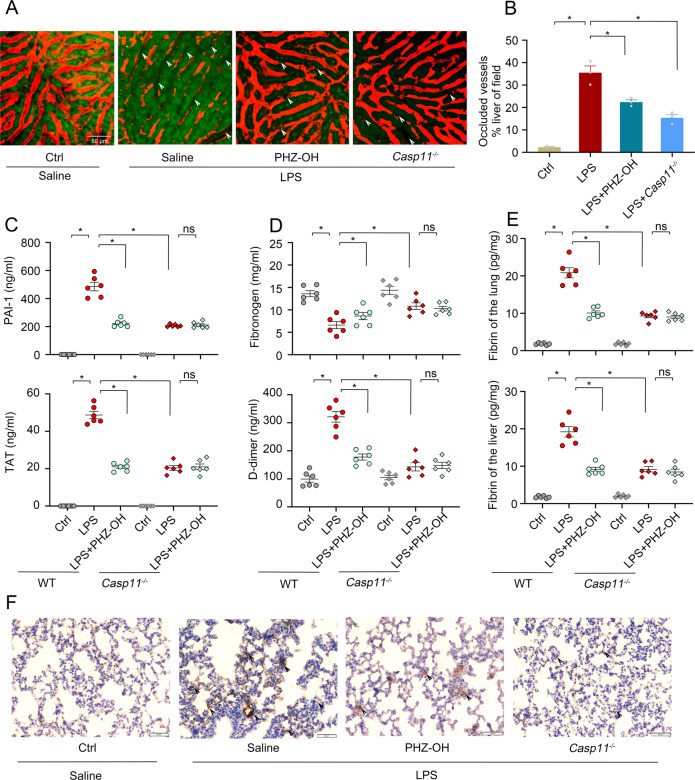
PHZ-OH inhibits coagulation and attenuates organ injury in mice challenged with LPS. WT or *Casp11*^*−/−*^ mice were primed with LPS (0.4 mg/kg) for 7 h prior to an intervention of PHZ-OH (5 mg/kg) and/or a challenge of LPS (4 mg/kg for 6 h for SD-IVM images or 10 mg/kg for 12 h for coagulation markers). **A** Representative SD-IVM images showing the circulating blood (Red) and the occlusion (as indicated by arrows) of the liver microvasculature that was clued by the signal of circulating blood (Red) and the autofluorescence of hepatocytes (Green); **B** Percent occluded region of the microvasculature related to the imaging field; Plasma concentrations of PAI-1 and TAT (**C**), and Fibrinogen and D-dimer (**D**); **E** Fibrin concentrations in the lung and the liver; **F** IHC staining showing the fibrin deposition in the lung (Dark brown, as indicated by arrows). Data are mean ± SEM of three mice for (**B**) and six mice for (**C**–**E**) in one experiment. *P < 0.05. ns, not significant. Scale bar = 50 μm.

We have previously revealed that caspase-11-dependent excessive coagulation activation would largely result in organ dysfunction and mortality in sepsis [22]. By using intravital microscopy, we herein visualized the microcirculation of hepatic sinusoid in intact and LPS-challenged mice. It was apparent that endotoxin (LPS) remarkably induced coagulation and subsequent vessel occlusion, and the administration of PHZ-OH significantly restored microcirculation to the level almost equivalent to that of *Casp11*-deficient mice (Fig. 5A, B). Besides, systematic coagulation activation dramatically augments the contents of thrombin-antithrombin (TAT) complex and plasminogen activator inhibitor type 1 (PAI-1), thereby consuming fibrinogen and increasing fibrinolysis and D-dimer, which are generally used as DIC markers [10, 22]. Almost in line with these illustrations, the plasma levels of TAT, PAI-1 and D-dimer, and the consumption of fibrinogen increased markedly in LPS-challenged mice when compared with the intact control (Fig. 5C, D). Notably, upon administration of PHZ-OH (5 mg/kg) to the mice under challenge of LPS, significant reductions of these DIC markers were observed in the WT mice, but not in *Casp11*-deficient mice (Fig. 5C, D). In addition, fibrin, the terminal executor of thrombosis in coagulation cascade, was determined by ELISA and immune-histochemical staining. Consistently, the LPS-boosted fibrin deposition in the lung and the liver was largely alleviated by the treatment of PHZ-OH (Fig. 5E, F). Taken together, these results indicate that PHZ-OH prevents coagulation and attenuates organ injury in sepsis via blocking caspase-11 pathway, suggesting that PHZ-OH may serve as a promising therapeutic molecule for the treatment of gram-negative bacterial sepsis.

### PHZ-OH suppresses LPS internalization and caspase-11-dependent TF activation

**Fig. 6:**
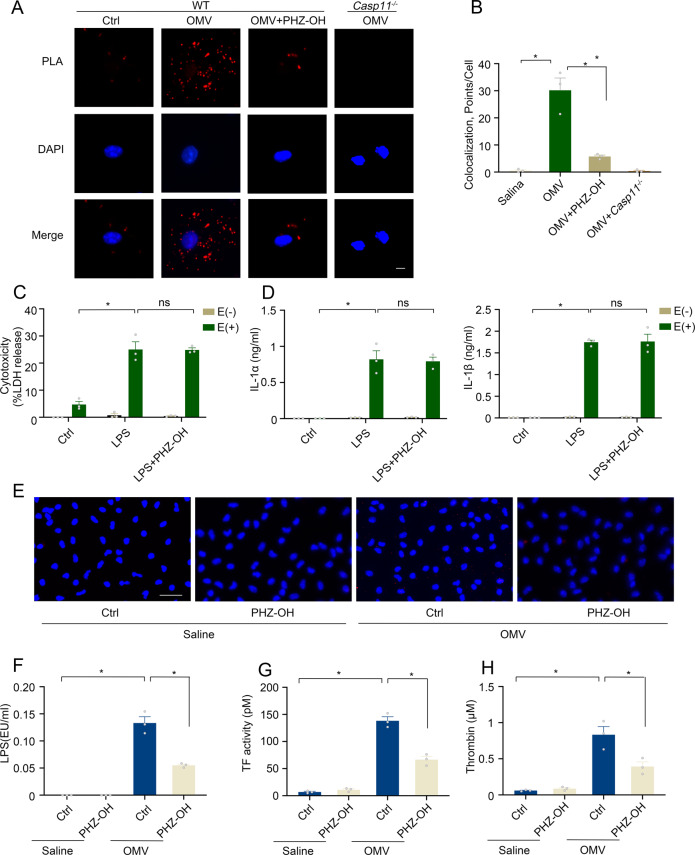
PHZ-OH inhibits LPS internalization and caspase-11-mediated TF activity. **A** Images of Proximity Ligation Assay (PLA) showing the accessing of LPS and caspase-11 (Red, Scar bar = 10 μm) within mouse peritoneal macrophages treated with saline or OMV (10 μg/ml) for 2 h in the presence or absence of PHZ-OH (4 μM); **B** Quantitative results of PLA. A spot means the average red points per cell in five random views of the indicated group within a slide; Cytotoxicity (**C**) and IL-1α/β release (**D**) of mouse peritoneal macrophages under a treatment of saline or PHZ-OH (4 μM) and/or a challenge of LPS (1 μg/ml) for 16 h with (E+) or without (E−) electroporation; Fluorescent staining of LPS (Red) (**E**) and LPS level of the cytosolic fraction assessed by using LAL assay (EU, endotoxin units) (**F**) in mouse peritoneal macrophages treated with saline or OMV (10 μg/mL) for 2 h in the presence or absence of PHZ-OH (4 μM); TF activity (**G**) and thrombin formation (**H**) of mouse peritoneal macrophages treated with saline or OMV (10 μg/mL) for 16 h in the presence or absence of PHZ-OH (4 μM). Data are shown as mean ± SEM of three independent experiments. *P < 0.05. ns, not significant.

As it is known that the accessing of LPS stimulates the oligomerization and activation of caspase-11, we next determined the colocalization of LPS and caspase-11 by using Proximity Ligation Assay (PLA), with the aim to investigate the mechanism by which PHZ-OH inhibits caspase-11 signaling. The results clearly indicated that PHZ-OH dampened the accessing of LPS to caspase-11 upon OMV stimulation when using *Casp11*-deficient macrophages as the negative control (Fig. 6A, B). To reveal if the effects of PHZ-OH involve LPS internalization, we directly delivered LPS into the cytosol of mouse macrophages by electroporation. Of note, PHZ-OH failed to inhibit the caspase-11-dependent cell death (Fig. 6C) and the release of IL-1α/β (Fig. 6D). Alternatively, it suppressed the OMV-mediated cytosolic level of LPS (Fig. 6E, F), suggesting that PHZ-OH inhibits caspase-11 pathway by blocking the cytosolic internalization of LPS rather than other signals. In addition, we have previously found that caspase-11-mediated pore-forming induces phosphatidylserine exposure, thus boosting tissue factor (TF) activity and triggering the extrinsic coagulation cascade that eventually mediates thrombin formation [6]. Consistent with our previous findings, PHZ-OH in the present study lowered down OMV-augmented TF activity as well as the formation of OMV-inflated thrombin (Fig. 6G, H). Collectively, PHZ-OH suppresses LPS internalization and thus prevents coagulation in sepsis by inhibiting caspase-11-boosted TF activity and the formation of thrombin.

### PHZ-OH inhibits LPS internalization by blocking AAK1-mediated endocytosis

**Fig. 7:**
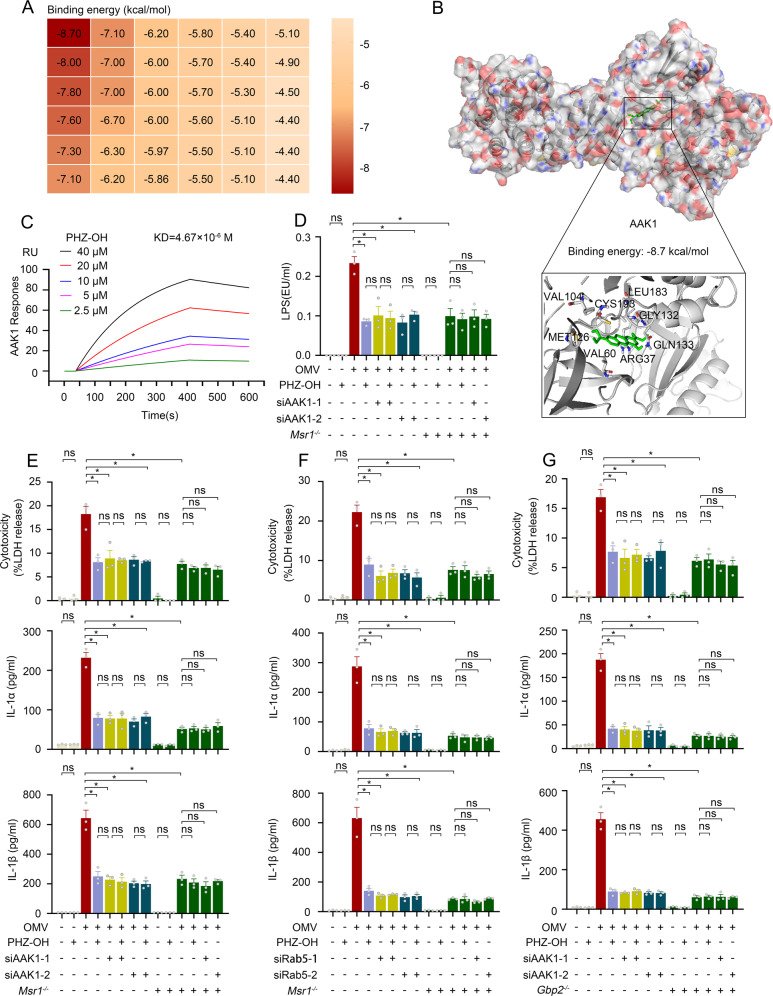
PHZ-OH inhibits LPS internalization by blocking AAK1-mediated endocytosis. **A** Binding energy of PHZ-OH with potential targets determined by using molecular docking [23]; **B** The computational 3D binding model between PHZ-OH and AP2-associated protein kinase 1 (AAK1); **C** The RU (response unit) vs. time in a sensorgram showing PHZ-OH (2.5, 5, 10, 20 and 40 μM) binding to the chip-immobilized AAK1 after subtracting the control signal (KD = 4.67 × 10^−6^ M); **D** LPS level of the cytosolic fraction assessed by using LAL assay in WT, AAK1-silenced, or *Msr1*-deficient macrophages challenged with OMV (10 μg/mL) for 2 h in the presence or absence of PHZ-OH (4 μM); **E** Cytotoxicity and IL-1α/β release of WT, AAK1-silenced, or *Msr1*-deficient macrophages treated with saline or OMV (10 μg/mL) for 16 h in the presence or absence of PHZ-OH (4 μM); **F** Cytotoxicity and IL-1α/β release of WT, Rab5-silenced, or *Msr1*-deficient macrophages treated with saline or OMV (10 μg/mL) for 16 h in the presence or absence of PHZ-OH (4 μM); **G** Cytotoxicity and IL-1α/β release of WT, AAK1-silenced, or *Gbp2*-deficient macrophages treated with saline or OMV (10 μg/mL) for 16 h in the presence or absence of PHZ-OH (4 μM). Data are shown as mean ± SEM of three independent experiments. *P < 0.05. ns, not significant.

It is well-documented that clathrin-dependent endocytosis functions in OMV-induced cytosolic LPS delivery [12]. To investigate the target of PHZ-OH in the inhibition of LPS internalization, we determined the binding of PHZ-OH with potential proteins involving LPS or clathrin-dependent endocytosis by using molecular docking, a computational approach for predicting the binding energy between the target and the ligands [23] (Fig. 7A and Table S2). We found that AP2-associated protein kinase 1 (AAK1), a key regulator of AP2 in clathrin-dependent endocytosis, showed highest binding energy (−8.7 kcal/mol) with PHZ-OH (Fig. 7A, B). In order to validate the physical binding of PHZ-OH and AAK1, we conducted surface plasmon resonance (SPR) [24] and discovered a high affinity between PHZ-OH and AAK1 (KD = 4.67 × 10^−6^ M, Fig. 7C). Furthermore, we verified that AAK1 knockdown (Fig. S8A) significantly inhibited OMV-augmented cytosolic LPS, whilst additional intervention of PHZ-OH onto AAK1-silenced macrophages did not display further effects (Fig. 7D), which was phenocopied in the levels of OMV-mediated cytotoxicity and IL-1α/β release (Fig. 7E). Given that macrophage scavenger receptor 1 (Msr1) mediates LPS internalization as shown in our previous study [22], we assessed the relationship of Msr1 and AAK1 in OMV-induced caspase-11 activation. It was observed that either administrating PHZ-OH or silencing AAK1 did not further decline OMV-augmented intracellular LPS and release of LDH and IL-1α/β on the basis of deleting Msr1 (Fig. 7D, E), indicating that Msr1 mediates cytosolic translocation of LPS via AAK1 signaling. As it is known that OMV delivers LPS into the cytosol via early endosome [12], we next knocked down Rab5 (Fig. S8B), an essential regulator of early endosome, in WT or *Msr1*-deficient macrophages. The results showed that knockdown of Rab5 significantly inhibited OMV-induced pyroptosis (Fig. 7F), almost in consistent with the reported findings [12]. Besides, similar to knockdown of AAK1, silence of Rab5 did not show additional effects on *Msr1*-deficient cells and PHZ-OH did not further affect Rab5-silenced cells in terms of OMV-induced cell death and cytokine release, implying Msr1-mediated endocytosis is involved in the process. Given that endosomal LPS accesses the cytosol under the assistance of guanylate-binding proteins (GBPs) [25], we conducted a similar experiment using WT or *Gbp2*-deficient cells (Fig. 7G). It was observed that GBP2 deletion, AAK1 knockdown and PHZ-OH administration alone had significant inhibition but did not display synergistic effects on OMV-induced pyroptosis (Fig. 7G). Taken together, these results indicate that Msr1/AAK1-dependent endocytosis is a pivotal signaling for regulating OMV-mediated cytosolic delivery of LPS and caspase-11 activation, and PHZ-OH is able to inhibit the LPS endocytosis via targeting AAK1.

### PHZ-OH attenuates endotoxin-induced coagulation and lethality by inhibiting AAK1

**Fig. 8:**
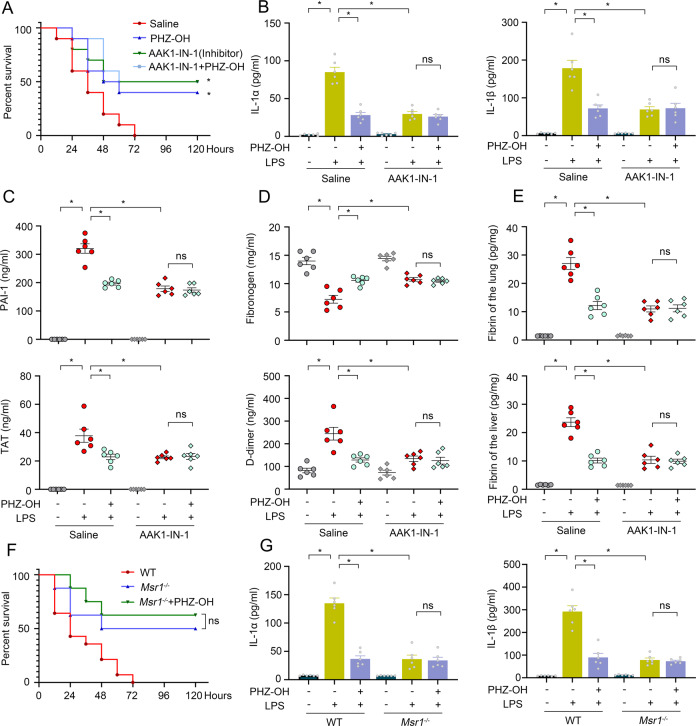
PHZ-OH alleviates endotoxin-induced coagulation and lethality by inhibiting AAK1. **A** Survival rate of WT mice treated with PHZ-OH (5 mg/kg) and/or AAK1-IN-1 (5 mg/kg) prior to a challenge of LPS (25 mg/kg); Plasma levels of IL-1α/β (**B**), PAI-1 and TAT (**C**), and fibrinogen and D-dimer (**D**), and fibrin concentrations of the lung and the liver (**E**) in WT mice primed with LPS (0.4 mg/kg) for 7 h prior to an intervention of PHZ-OH (5 mg/kg) and/or a challenge of LPS (10 mg/kg) for 12 h. **F** Survival rate of WT and *Msr1*^*−/−*^ mice treated with or without PHZ-OH (5 mg/kg) prior to a challenge of LPS (25 mg/kg); **G** Plasma IL-1α/β levels of saline-treated or LPS-challenged (0.4 mg/kg LPS for 7 h and a following 10 mg/kg LPS for 12 h) WT and *Msr1*^*−/−*^ mice under an intervention of PHZ-OH (5 mg/kg) or not. Data are mean ± SEM of six mice in one experiment. *P < 0.05. ns, not significant.

To investigate the inhibitory effect of PHZ-OH on AAK1 in in-vivo study, we treated LPS-challenged mice with PHZ-OH and/or AAK1-IN-1 (a well-known AAK1 inhibitor). Either PHZ-OH or AAK1-IN-1 alone significantly improved the survival rate of mice and reduced caspase-11-related cytokines in endotoxemia model, whilst PHZ-OH plus AAK1-IN-1 did not display any additional improvement and reduction, respectively (Fig. 8A, B). Moreover, LPS-inflated coagulation levels, such as increase of PAI-1, TAT and D-dimer, consumption of fibrinogen, and augment of fibrin in the lung and the liver, were significantly dropped when administrating PHZ-OH or AAK1-IN-1 (Fig. 8C–E). Nevertheless, PHZ-OH and AAK1-IN-1 did not have synergistic effects on the inhibition of coagulation (Fig. 8C–E). Further in-vivo study using *Msr1*^*−/−*^ mice also indicated that administration of PHZ-OH did not significantly affect LPS-induced mortality and augment of IL-1α/β and coagulation at the basis of Msr1 depletion (Fig. 8F, G). These results thus suggest that Msr1/AAK1 is essential for endotoxin-induced coagulation and death, and PHZ-OH can suppress the complications through targeting AAK1.

## Discussion

Caspase-11, as an intracellular LPS sensor of innate immune system, is not only able to mediate GSDMD-dependent cell death, but also can trigger NLRP3 inflammasome, thereby inducing the release of inflammatory cytokines independent or dependent of the inflammasome [18]. Our previous study has revealed the essential role of NLRP3 inflammasome for clearing invading pathogens in moderate infection [26]. Nevertheless, caspase-11 is reported to be different from caspase-1 in the defense of infection [27]. In addition, overactivation of caspase-11, commonly caused by severe bacterial infection, can induce organ dysfunction and host death in sepsis [3–5]. We have previously found that blocking caspase-11 genetically and pharmacologically significantly reduces sepsis-induced organ injury and lethality, and thus may be a promising strategy for the treatment of sepsis [6, 28]. Consistently, PHZ-OH, a novel 1-hydroxy phenothiazinium-based small molecule identified in this study, significantly prevents coagulation, organ dysfunction and death in sepsis by inhibiting caspase-11 signaling rather than NLRP3 inflammasome. Furthermore, the inhibition effect of PHZ-OH does not affect global inflammation, including the release of TNFα and IL-6. The selective inhibition may allow the maintenance of host response to secondary infection and avoid immunosuppression. Although inflammatory cytokine storm is commonly accompanied by organ injury or even death in sepsis, targeting inflammation therapy remains questionable in clinical applications [29, 30]. It has been proved that anti-TNFα antibody or antagonists of TLR4 fails to decline the mortality of septic patients [31–33]. Thus, PHZ-OH, with selectively blocking caspase-11 signaling but not the global inflammation, would be an alternative and favorable medicine candidate for rescuing sepsis.

We recently bridged caspase-11 and coagulation activation independent of inflammatory response [6]. In the present study, PHZ-OH demonstrates the ability to prevent coagulation by inhibiting caspase-11 signaling. Given that overactivation of coagulation and the emergence of DIC would be the major cause of organ injury and death in sepsis [2, 8, 9], anticoagulation therapeutics were previously brought into clinical trials for treating septic patients with DIC. However, administration of anticoagulant agents displays a risk of bleeding and fails to improve the prognosis of patients with sepsis [2, 34, 35]. In the present study, PHZ-OH blocks the key pathway that initiates the blood coagulation cascade but not the cascade itself. This strategy may provide an alternative option to rescue coagulation syndrome and improve the prognosis of sepsis.

Mechanistically, we discovered that Msr1/AAK1-dependent endocytosis essentially functions in OMV-mediated LPS internalization. Internalization of LPS by endocytosis is firstly required for caspase-11 activation in the sensing process [12]. AAK1 recruits AP2 to cell membrane and phosphorylates the threonine residue of the µ–subunit (AP2M1), which enhances the binding of AP2 with cargo and increases clathrin-dependent endocytosis [36]. PHZ-OH inhibits AAK1-mediated LPS internalization and leaves the function of caspase-11 intact, which does not affect the physiological function of caspase-11 in the protection against cytosolic bacteria and the surveillance of immune cells [37]. Thus, the selective inhibition effect of PHZ-OH on caspase-11 signaling may have fewer side effects, and inhibition of LPS internalization by targeting AAK1 would be a promising strategy in the treatment of bacterial sepsis. In addition, a recent study suggested that baricitinib, approved for treating rheumatoid arthritis, may serve as an anti-SARS-CoV-2 drug by inhibiting AAK1-regulated virus endocytosis. Thus, AAK1 would be a hopeful anti-SARS-CoV-2 target and PHZ-OH may be a new potential candidate for drug development to treat SARS-CoV-2, which remains to be investigated in further study.

In addition to the inhibition effect on caspase-11-mediated coagulation, PHZ-OH also demonstrates excellent dose-dependent antibacterial activity, which may further improve therapeutic effects for the treatment of sepsis, especially in the current era of rapid emergence of multi-drug-resistant bacteria. Taken together, our study for the first time discovered a novel 1-hydroxy phenothiazinium-based molecule (PHZ-OH), capable of preventing bacterial sepsis by selectively inhibiting AAK1-mediated LPS internalization for caspase-11 activation, which may provide a novel target and a potent therapeutic molecule in the treatment of bacterial sepsis.

## Methods and materials

### Reagents

General chemical reagents were purchased from commercial suppliers (Acros/Aldrich Chemical Co. Ltd, USA, J & K Scientific Ltd, China) and used without further purification, unless otherwise noted. LDH Assay kits were from Beyotime Biotechnology (Cat: C0017). Commercial ELISA kits including mouse IL-1α (Cat: 88-5019, Invitrogen), mouse IL-1β (Cat: 88-7013, Invitrogen), mouse TNF (Cat: 88-7324, Invitrogen), mouse IL-6 (Cat: 88-7064, Invitrogen) human IL-1α (DY200, R&D), human IL-1β (Cat: 88-7261, Invitrogen), mouse D-dimer (Cat: CEA506Mu, Cloud-Clone), mouse fibrinogen (Cat: ab108844, Abcam), mouse PAI-1 (Cat: ab197752, Abcam), mouse TAT (Cat: ab137994, Abcam) and mouse fibrin (MBS164817, MybioSource) were purchased from commercial suppliers as indicated. Human Tissue Factor Chromogenic Activity Assay kit (Cat: CT1002b) and SensoLyte internally quenched 5-FAM/QXL-520 FRET thrombin substrate (Cat: AS-72129, Anaspec) were obtained to detect TF activity and thrombin formation, respectively. Antibodies used in this study, including anti-caspase-11 (Cat: C1354, Sigma), anti-mouse GSDMD (Cat: ab209845, Abcam), anti-mouse IL-1α (Cat: ab7632, Abcam), anti-mouse IL-1β (Cat: AF-401-NA, R&D system) and anti-mouse β-actin (clone 8H10D10, Cat: 3700S, Cell Signaling Technologies), were commercially obtained except anti-mouse fibrin antibody (59D8)–a gift from Prof. Nigel Mackman. AAK-IN-1 (E0498) was purchased from Selleck, and siRNAs were ordered from RiboBio. Nigericin (tlrl-nig-5) and Ultra-LPS (tlrl-pb5lps) were purchased from InvivoGen, whilst LPS derived from *E. coli* 0111:B4 was from Sigma (L2630). OMV was collected and purified as previously described [12]. In brief, *E. coli* (ATCC, 25922) was cultured in 5 × 200 ml of LB at 37 °C and 220 rpm until OD_600_ of 0.5 was achieved. Supernatant was harvested after a centrifuge at 10, 000 g for 10 min at 4 °C, followed by a filtration through a 0.45 μm filter to remove bacteria and debris. OMVs were then isolated by ultracentrifugation at 400,000 × *g* for 1.5 h at 4 °C in a Beckman opima L-100 XP rotor and resuspended in 1.5 ml of sterile ultrapure PBS. The protein level was assessed by using Pierce BCA protein assay kit (Thermo Scientific) and a final filtration was conducted via a 0.45 μm filter before further applications.

### Mice and models of endotoxemia and bacterial sepsis

*Casp11*^−/−^ (Jackson Laboratory, 024698), *Casp1*^−/−^ (Jackson Laboratory, 032662), *Casp1/11*^−/−^ (Jackson Laboratory, 016621), *Nlrp3*^*−/−*^ mice (Jackson Laboratory, 021302), *HCV* mice (express human tissue factor, Nigel Mackman Lab), *Myd88*^*−/−*^ (Jackson Laboratory, 009088), *Gbp2−/−* (GemPharmatech Co., Ltd, T028952), *Msr1*^*−/−*^ (GemPharmatech Co., Ltd, T014075) and WT male C57BL/6J mice, with age of 8–10 weeks and body weight of 22-25 g, were randomly allocated and used in in-vivo study without blinding. Mice were treated with saline or chemicals for 30 min before or after a challenge of cecal ligation and puncture (CLP), 10^9^ CFU *E. coli* (ATCC, 25922) or 25 mg/kg LPS. Survival rates (around 10 mice per group) were observed for 7 days, whilst the tissues or the plasma were obtained at 12 h after challenges (6 mice per group). To generate DIC-like model (6 mice per group), mice were primed with 0.4 mg/kg LPS for 7 h and challenged with 10 mg/kg LPS [3, 4, 6]. Chemicals were intraperitoneally injected 30 min prior to the challenge of 10 mg/kg LPS. In order to visualize most of the microvascular that are not completely occluded under intravital miscroscopy (3 mice per group), 4 mg/kg of LPS after priming were intraperitoneally injected to mice 6 h prior to imaging [6, 10]. Standard condition at room temperature and a 12-h light–dark cycle were provided to all mice that were free to access to water and standard chow. Animal experiments were carried out in compliance with the guidelines by the committee of Xiangya Hospital and Central South University.

### Cecal ligation and puncture (CLP)

After anesthetized with 2% isoflurance (Piramal Critical Care) in oxygen, mice were subjected to a 1.5 cm longitudinal midline incision. The cecum was exposed and ligated (75%) prior to a through-and-through puncture and an extrusion of a small amount feces, followed by a relocation of the cecum and a close of the abdomen. Recovery was allowed by injecting pre-warmed saline (1 mL per mouse) in a 37 °C warmer. Sham-operated mice were subjected to the same surgery except the ligation and the puncture.

### Intravital microscopy and image analysis

To real-time visualize the circulation of microvasculature, the left liver of mice was exposed and externalized on a thin glass coverslip after anesthetized with xylazine hydrochloride (10 mg/kg) and ketamine hydrochloride (200 mg/kg). The glass coverslip was then loaded on the thermal control stage of the inverted microscope of the confocal intravital microscopy, followed by injecting AF647-conjugated anti-mouse albumin antibody (0.05 μg/mouse) via the right jugular vein. Fluorescent images were acquired and the analysis was conducted as previously described [6, 10].

### Cell culture

As previously described [6, 10], mouse peritoneal macrophages were obtained and purified from *Casp11*^−/−^, HCV (human TF positive), *Msr1*^−/−^ or WT C57BL/6J male mice. The cells were plated overnight prior to the treatment of siRNA (Ribobio, China) or chemicals (1–10 μM) and/or the challenge of OMV (10 μg/mL). In addition, THP-1 cells were plated and treated with 20 ng/mL of PMA (Sigma) overnight. Interventions of chemicals (1–4 μM) were given when the cells were challenged with OMV (10 μg/mL).

### Proximity ligation assay

Proximity Ligation Assay kit (PLA, Sigma) was utilized to determine the interaction of LPS and caspase-11. Briefly, mouse peritoneal macrophages on glass slides were treated with chemicals and/or challenged with OMV (10 μg/mL) for 2 h. After fixed with 4% formaldehyde and permeabilized by PBS containing 1% Triton X-100, the macrophages were incubated with primary antibodies to LPS (mouse monoclonal 2D7/1, Abcam, ab35654) or caspase-11 (rat monoclonal 17D9, Sigma-Aldrich, C1354) at 4 °C overnight. PLA was performed in situ according to the instructions of the manufacture. Images were obtained by using Nikon confocal laser scanning microscope and analyzed by using ImageJ software.

### Cytosolic LPS Determination

Cytosolic LPS was visualized by fluorescent staining and quantified by using Limulus Amebocyte Lysate (LAL) assay. In brief, mouse peritoneal macrophages in various treatments were challenged with 10 μg/mL OMV for 2 h. After washed with sterile cold PBS four times on a platform shaker on ice, the cells on glass slides or wells were applied for staining or LAL assay respectively. For LPS staining, the cells were fixed with 4% formaldehyde and permeabilized by PBS containing 1% Triton X-100, followed by incubations of a primary anti-LPS antibody at 4 °C overnight and a secondary Cy3-conjugated goat anti-mouse IgG antibody (Cat#: SA00009-1, Proteintech). Four times of washes using PBST were conducted between steps. Fluorescent signals were visualized by using Nikon confocal laser scanning microscope after DAPI staining. For the quantity of cytosolic LPS, 4 × 10^6^ cells per group were treated with 300 µl of 0.005% digitonin extraction buffer on ice for 10 min [12], and the supernatant (cytosolic fraction) was obtained and applied to LAL assay according to the manufacturer’s introduction.

### Tissue histology

Mice were sacrificed and perfused using cold phosphate-buffered saline (PBS) containing heparin (20 IU/mL) and subsequent 10% formalin. After further fixed with 10% formalin overnight, the lung was embedded in paraffin and then split into 4 μm of sections for hematoxylin and eosin (H&E) staining and immunohistochemistry (IHC). The sections in IHC were incubated with primary antibody (anti-mouse fibrin antibody, 1:500) at 4 °C overnight after dewaxing, followed by an incubation of the secondary antibody (anti-mouse HRP-conjugated antibody, 1:2000) at RT for 2 h. Positive staining of the sections was finally visualized by adding 3, 3′-diaminobenzidine tetrahydrochloride and imaged by using Nikon microscopy. PBST (PBS containing 0.1% tween-20) was used for four times of washes between steps.

### qRT-PCR

After extracted from macrophages using Trizol Reagent (Invitrogen, USA), total RNA was reversely transcribed into complementary DNA using a Transcriptor First Strand cDNA Synthesis Kit (Invitrogen, USA). Caspase-11 mRNA expression was determined by quantitative real-time polymerase chain reaction (qRT-PCR) using qPCR mix (Vazymebiotech, China) and designed primers on a LightCycler 480II analyzer (Roche, Mannheim, Germany). The primer sequences were: 5′-ACAAACACCCTGACAAACCAC-3′ and 5′-CACTGCGTTCAGCATTGTTAAA-3′ for caspase-11, 5′-GACAACAGCCAAAAGTTCAGACC-3′ and 5′-CCAAAGACTGCACTGTGGGTT-3′ for AAK1, 5′-GCTAATCGAGGAGCAACAAGAC-3′ and 5′-CCAGGCTTGATTTGCCAACAG-3′ for Rab5, and 5′-AGGTCGGTGTGAACGGATTTG-3′ and 5′-TGTAGACCATGTAGTTGAGGTCAGAPDH-3′ for GAPDH, respectively. Fold change of caspase-11 was expressed as the 2−ΔΔCt relative to negative control group after the normalization by using GAPDH.

### Western blot

SDS-polyacrylamide gel electrophoresis was used to separate proteins that were subsequently transferred to PVDF membranes. After a block using 5% fat-free milk, the membranes were incubated with primary antibodies at 4 °C overnight and a following HRP-conjugated secondary antibody at RT for 2 h. Four times of washes using TBST were conducted between steps. Western Bright ECL-Spray (advansta, catalog number: K-12049-D50) was added onto the membrane and the blots were visualized using Bio-Rad system.

### TF activity and thrombin assay

After plated in 96-well plates, human TF-positive macrophages were treated with chemicals and OMV. After 3 times of washes with PBS, the cells were incubated with 70 μL assay mixture (containing factor VII and factor X) at 37 °C for 30 min, followed by addition of Factor Xa substrate. The absorbance at 405 nm was determined and the TF activity was calculated according to the standard absorbance curve. Thrombin generation assay was used to assess thrombin formation [6]. Briefly, the mixture containing 10 μL cell supernatant and diluted platelet poor plasma was subjected to 50 μL thrombin substrate. The fluorescence intensity was detected by using TECAN multi-functional fluorescence microplate reader.

### Molecular docking and surface plasmon resonance

Molecular docking, a computational approach to calculate the binding energy between proteins and ligands [23], were used for screening the target of PHZ-OH. Given that OMV mediates cytosolic delivery of LPS by clathrin-dependent endocytosis [12], the crystal structures of potential targets, including LPS-related receptors or endocytosis-associated proteins, were retrieved from http://www.rcsb.org/ (Table S2), while the structural formulas of PHZ-OH was constructed using Chim3D (2010). Molecular docking between PHZ-OH and proteins was conducted by using AutoDock 4.0 and AutoDock Tools 1.5.6, in which independent docking calculation was conducted with 250,0000 evaluations using Lamarckian Genetic Algorithm. To validate the binding of PHZ-OH and AAK1 in physics, surface plasmon resonance was subsequently performed by using BIAcore4000 (BIAcore). The equilibrium-binding constant (KD, the lower value the higher affinity) of PHZ-OH and AAK1 was determined as described previously [24].

### Statistical analysis

Statistical analysis was conducted with GraphPad Prism 7.0 software using two-tailed methods by a blinded operator. One-way or Two-way ANOVA with post-hoc test was used for comparisons between multiple groups when data tests were assumed (e.g., normal distribution; variance similarity). The survival rates were analyzed using the log-rank test. *P* values less than 0.05 were considered as statistical significance. None of the samples or animals were excluded from the statistical analysis. Sample sizes referred to the general application of the field and were not statistically predicted.

## Supplementary information

Supporting Information

Uncropped WB images

Reproducibility checklist

## Data Availability

The datasets used and/or analyzed during the current study are available from the corresponding author on reasonable request.

## References

[1] Stevenson EK, Rubenstein AR, Radin GT, Wiener RS, Walkey AJ (2014). Two decades of mortality trends among patients with severe sepsis: a comparative meta-analysis. Crit Care Med.

[2] Gando S, Levi M, Toh CH (2016). Disseminated intravascular coagulation. Nat Rev Dis Prim.

[3] Hagar JA, Powell DA, Aachoui Y, Ernst RK, Miao EA (2013). Cytoplasmic LPS activates caspase-11: implications in TLR4-independent endotoxic shock. Science.

[4] Kayagaki N, Wong MT, Stowe IB, Ramani SR, Gonzalez LC, Akashi-Takamura S (2013). Noncanonical inflammasome activation by intracellular LPS independent of TLR4. Science.

[5] Shi J, Zhao Y, Wang Y, Gao W, Ding J, Li P (2014). Inflammatory caspases are innate immune receptors for intracellular LPS. Nature.

[6] Yang X, Cheng X, Tang Y, Qiu X, Wang Y, Kang H (2019). Bacterial endotoxin activates the coagulation cascade through gasdermin D-dependent phosphatidylserine exposure. Immunity.

[7] Engelmann B, Massberg S (2013). Thrombosis as an intravascular effector of innate immunity. Nat Rev Immunol.

[8] Spero JA, Lewis JH, Hasiba U (1980). Disseminated intravascular coagulation. Findings in 346 patients. Thrombosis Haemost.

[9] Levi M, Ten Cate H (1999). Disseminated intravascular coagulation. N Engl J Med.

[10] Yang X, Cheng X, Tang Y, Qiu X, Wang Z, Fu G (2020). The role of type 1 interferons in coagulation induced by gram-negative bacteria. Blood.

[11] Blot S, Antonelli M, Arvaniti K, Blot K, Creagh-Brown B, de Lange D (2019). Epidemiology of intra-abdominal infection and sepsis in critically ill patients: “AbSeS”, a multinational observational cohort study and ESICM Trials Group Project. Intensive Care Med.

[12] Vanaja SK, Russo AJ, Behl B, Banerjee I, Yankova M, Deshmukh SD (2016). Bacterial outer membrane vesicles mediate cytosolic localization of LPS and caspase-11 activation. Cell.

[13] Xiao Q, Lin H, Wu J, Pang X, Zhou Q, Jiang Y (2020). Pyridine-embedded phenothiazinium dyes as lysosome-targeted photosensitizers for highly efficient photodynamic antitumor therapy. J Med Chem.

[14] Hu Z, Xiao Q, Xiao D, Wang Z, Gui F, Lei Y (2021). Synthesis of anti-poisoning spinel Mn-Co-C as cathode catalysts for low-temperature anion exchange membrane direct ammonia fuel cells. ACS Appl Mater Interfaces.

[15] Lipinski CA, Lombardo F, Dominy BW, Feeney PJ (2001). Experimental and computational approaches to estimate solubility and permeability in drug discovery and development settings. Adv Drug Deliv Rev.

[16] Hann MM, Oprea TI (2004). Pursuing the leadlikeness concept in pharmaceutical research. Curr Opin Chem Biol.

[17] Veber DF, Johnson SR, Cheng HY, Smith BR, Ward KW, Kopple KD (2002). Molecular properties that influence the oral bioavailability of drug candidates. J Med Chem.

[18] Kayagaki N, Warming S, Lamkanfi M, Vande Walle L, Louie S, Dong J (2011). Non-canonical inflammasome activation targets caspase-11. Nature.

[19] Deng M, Tang Y, Li W, Wang X, Zhang R, Zhang X (2018). The endotoxin delivery protein HMGB1 mediates caspase-11-dependent lethality in sepsis. Immunity.

[20] Phoenix DA, Harris F (2003). Phenothiazinium-based photosensitizers: antibacterials of the future?. Trends Mol Med.

[21] Xiao Q, Mai B, Nie Y, Yuan C, Xiang M, Shi Z (2021). In vitro and in vivo demonstration of ultraefficient and broad-spectrum antibacterial agents for photodynamic antibacterial chemotherapy. ACS Appl Mater Interfaces.

[22] Yuan C, Wu M, Xiao Q, Zhao W, Li H, Zhong Y (2021). Blocking Msr1 by berberine alkaloids inhibits caspase-11-dependent coagulation in bacterial sepsis. Signal Transduct Target Ther.

[23] Sarnpitak P, Mujumdar P, Taylor P, Cross M, Coster MJ, Gorse AD (2015). Panel docking of small-molecule libraries - Prospects to improve efficiency of lead compound discovery. Biotechnol Adv.

[24] Aćimović SS, Ortega MA, Sanz V, Berthelot J, Garcia-Cordero JL, Renger J (2014). LSPR chip for parallel, rapid, and sensitive detection of cancer markers in serum. Nano Lett.

[25] Finethy R, Luoma S, Orench-Rivera N, Feeley EM, Haldar AK, Yamamoto M (2017). Inflammasome activation by bacterial outer membrane vesicles requires guanylate binding proteins. mBio.

[26] Zhong Y, Lu Y, Yang X, Tang Y, Zhao K, Yuan C (2020). The roles of NLRP3 inflammasome in bacterial infection. Mol Immunol.

[27] Broz P, Ruby T, Belhocine K, Bouley DM, Kayagaki N, Dixit VM (2012). Caspase-11 increases susceptibility to Salmonella infection in the absence of caspase-1. Nature.

[28] Tang Y, Wang X, Li Z, He Z, Yang X, Cheng X (2021). Heparin prevents caspase-11-dependent septic lethality independent of anticoagulant properties. Immunity.

[29] Cohen J (2002). The immunopathogenesis of sepsis. Nature.

[30] Deng M, Scott MJ, Loughran P, Gibson G, Sodhi C, Watkins S (2013). Lipopolysaccharide clearance, bacterial clearance, and systemic inflammatory responses are regulated by cell type-specific functions of TLR4 during sepsis. J Immunol.

[31] Abraham E, Anzueto A, Gutierrez G, Tessler S, San Pedro G, Wunderink R (1998). Double-blind randomised controlled trial of monoclonal antibody to human tumour necrosis factor in treatment of septic shock. NORASEPT II Study Group. Lancet.

[32] Opal SM, Laterre PF, Francois B, LaRosa SP, Angus DC, Mira JP (2013). Effect of eritoran, an antagonist of MD2-TLR4, on mortality in patients with severe sepsis: the ACCESS randomized trial. JAMA.

[33] Rice TW, Wheeler AP, Bernard GR, Vincent JL, Angus DC, Aikawa N (2010). A randomized, double-blind, placebo-controlled trial of TAK-242 for the treatment of severe sepsis. Crit Care Med.

[34] Warren BL, Eid A, Singer P, Pillay SS, Carl P, Novak I (2001). Caring for the critically ill patient. High-dose antithrombin III in severe sepsis: a randomized controlled trial. JAMA.

[35] Kienast J, Juers M, Wiedermann CJ, Hoffmann JN, Ostermann H, Strauss R (2006). Treatment effects of high-dose antithrombin without concomitant heparin in patients with severe sepsis with or without disseminated intravascular coagulation. J Thrombosis Haemost.

[36] Ricotta D, Conner SD, Schmid SL, von Figura K, Honing S (2002). Phosphorylation of the AP2 mu subunit by AAK1 mediates high affinity binding to membrane protein sorting signals. J Cell Biol.

[37] Aachoui Y, Leaf IA, Hagar JA, Fontana MF, Campos CG, Zak DE (2013). Caspase-11 protects against bacteria that escape the vacuole. Science.

