# Supplementary material for: Novel 1-hydroxy phenothiazinium-based derivative protects against bacterial sepsis by inhibiting AAK1-mediated LPS internalization and caspase-11 signaling
Source: Cell Death Dis. 2022 Aug 18;13(8):722. doi: 10.1038/s41419-022-05151-7 (PMC9387894; doi:10.1038/s41419-022-05151-7)
Supplement: Supplementary file 1 — Supporting Information [file 41419_2022_5151_MOESM1_ESM.pdf]

Supporting Information

Contents:

01. General information…………………………………………………… S2
02. Chemistry and synthesis……………………………………………… S2
03. Scheme S1….……………………………………………………… S2
04. Table S1..………………………………………………………… S3
05. Appendix…………………………………………………………… S4
06. Figure S1…………………………………………………………… S4
07. Figure S2…………………………………………………………… S5
08. Figure S3…………………………………………………………… S6
09. Figure S4…………………………………………………………… S7
10. Figure S5…………………………………………………………… S8
11. Figure S6…………………………………………………………… S9
12. Figure S7…………………………………………………………… S10
13. Figure S8…………………………………………………………… S11
14. Figure S9…………………………………………………………… S11
14. Table S2..…………………………………………………………… S12

1. General information

Unless otherwise noted, all reagents were purchased from commercial vendors and used without further treatments. All moisture-sensitive reactions were carried out in dried solvents under a nitrogen atmosphere. Nuclear magnetic resonance (NMR) spectra were recorded at 400 MHz (Bruker Avance III NMR Spectrometer) for ^{1}H NMR and 100 MHz for ^{13}C NMR. Mass spectra (MS) were performed on a Waters ACQUITY UPLC M-Class spectrometer and High Resolution Mass Spectra (HRMS) were acquired on a Thermo Finnigan MAT 95 KL mass spectrometer. High-performance liquid chromatography (HPLC) chromatograms were acquired a Waters ACQUITY Arc system with a GL Sciences Inertsil ODS-4 column (150 mm × 4.6 mm, 5 μm) and an auto-sampler.

2. Synthesis and Characterization

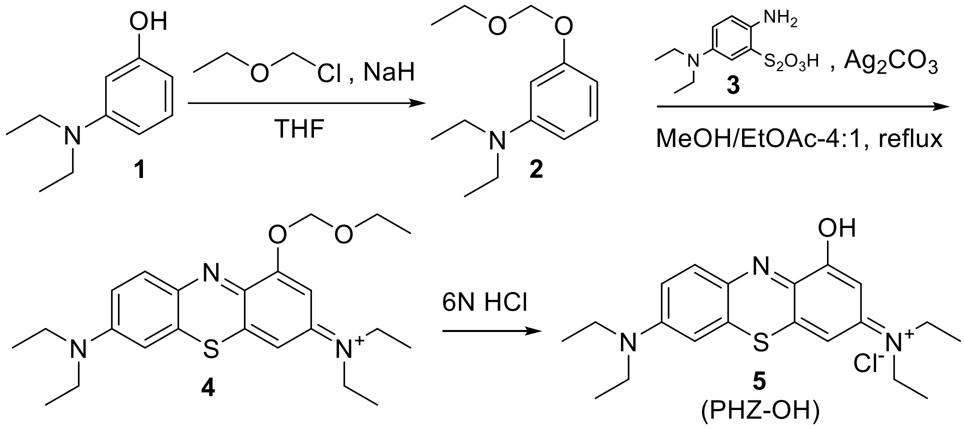

Scheme S1. Schematic Illustration for the preparation of compound **5** (PHZ-OH)

3-(ethoxymethoxy)-*N*,*N*-diethylaniline (2)

To a stirred solution of 3-(diethylamino)phenol (compound **1**, 1.012 g, 6 mmol) in 20 mL dried THF was added NaH (0.36 g, 9 mmol) at 0 °C, the reaction mixture was stirred in an ice-box for 30 min. Then (chloromethoxy)ethane (0.7 mL, 7.2 mmol) was added slowly with stirring. The reaction mixture was warmed up to room temperature and stirred for another 6 h, then a solution of saturated NH_4Cl (3 mL) was added to quench the reaction. After removal of the solvent, the mixture was re-dissolved in 20 mL ethyl acetate, washed by saturated brine, and then the organic layer was dried and concentrated to leave oil. The residue was purified by flash chromatography with diethyl ether/hexane as eluent to afford compound **2** as a colorless oil (0.91 g, 68%). ESI-MS: $[M+H]^+$ 224.2. ^{1}H NMR (400 MHz, $CDCl_3$): δ 7.16-7.11 (m, 1H), 6.40-6.36 (m, 3H), 5.24 (s, 2H), 3.77 (q, J = 7.1 Hz, 2H), 3.66 (q, J = 7.1 Hz, 4H), 1.26 (t, J = 7.1 Hz, 3H), 1.19 (t, J = 7.1 Hz, 6H); ^{13}C NMR (100 MHz, $CDCl_3$): δ 159.0, 149.2, 130.0, 105.9, 102.8, 100.3, 93.3, 64.1, 44.5, 15.2, 12.7 ppm.

2-amino-5-(diethylamino)benzenesulfonoperoxothioic O-acid (3) (ref)

Compound **3** was prepared as reported. Briefly, To a solution of *N*,*N*-diethyl-*p*-phenylenediamine (1 eq) in water and methanol (H_2O/MeOH 4:1) was added $ZnCl_2$ (6.3 M in water, 1.0 eq) and HCl (10 mol/L, 1 eq). Then the mixture was cooled to 0 °C with an ice-water box, and an aqueous solution of newly prepared $K_2Cr_2O_7$ (0.5 mol/L, 3.0 eq) and $Na_2S_2O_3$ (3 mol/L, 2.0 eq) were added, and the mixture was stirred for another 3 h. After warmed up to room temperature (RT), the reaction mixture was stirred for a further 1 h, and then the mixture was filtered, washed with water and acetone, and

dried under vacuum to afford compound **3** as gray solid, which was used as such in the next step.

3,7-bis(diethylamino)-1-hydroxyphenothiazin-5-ium chloride (5)

Compounds **2** (55.8 mg, 0.25 mmol) and **3** (0.14 g, 0.5 mmol) were dissolved in methanol and ethyl acetate (6 mL, MeOH/Ethyl Acetate 4:1), and the mixture was stirred at 80 °C for a while. Silver carbonate (0.14 g, 0.5 mmol) was then added slowly under reflux to generate a deep blue solution. The reaction mixture was refluxed with stirring for 2 h, and then the mixture was allowed to cool to RT and filtered by celite, and next the solid was washed by methanol and DCM. The filtrate was concentrated to leave a residue, and purified by flash chromatography to afford a deep blue solid, which was used as such in the next step.

To a stirred solution of the above obtained compound **4** (40.0 mg, 0.094 mmol) in a dried solution of DCM and THF (6 mL, 1:1) was added HCl (0.1 mL, 5N in MeOH), and the mixture was refluxed for 2 h. After cooled to room temperature, the solvent was removed under reduced pressure, and the residue was purified by flash chromatography to afford compound **5** as a blue solid (31.0 mg, 35% for 2 steps). ESI-MS: $[M\text{-}Cl]^+$ 356.2; ESI-HRMS: $[M\text{-}Cl^-]^+$ calcd 356.1791; found 356.1791. ^1H NMR (400 MHz, MeOD): δ 7.95 (d, J = 9.6 Hz, 1H), 7.31 (dd, 9.5, 2.5 Hz, 1H), 7.20 (d, J = 2.5 Hz, 1H), 7.02 (d, J = 2.3 Hz, 1H), 6.68 (d, J = 2.4 Hz, 1H), 3.71 (q, J = 7.0 Hz, 8H), 1.35-1.30 (m, 12H); ^{13}C NMR (100 MHz, MeOD): δ 162.4 (C), 157.1 (C), 153.9 (C), 139.2 (CH), 135.8 (C), 135.2 (C), 133.7 (C), 130.1 (C), 118.2 (CH), 107.0 (CH), 103.2 (CH), 99.6(CH), 47.5 (CH_2), 47.0 (CH_2), 13.3 (CH_3), 13.0 (CH_3) ppm.

Table S1 HPLC and HRMS data for compound **5**.

Compound	t_R (min)[a]	Purity	MS calcd	HRMS[b]
**5**	15.13	99%	356.17911	356.17915

[a] Analytical HPLC method: GL Sciences Inertsil ODS-4 column (150 mm × 4.6 mm，5 μm); solvent system: A, MeCN; B, 0.1% TFA in water; A from 30%-100% over 30 min; flow rate, 1.0 mL/min; wavelength 254 nm; injection volume 10 μL, column temperature, 30 °C. [b] Purified products were further characterized by high resolution mass spectrometry (HRMS).

4. Appendix

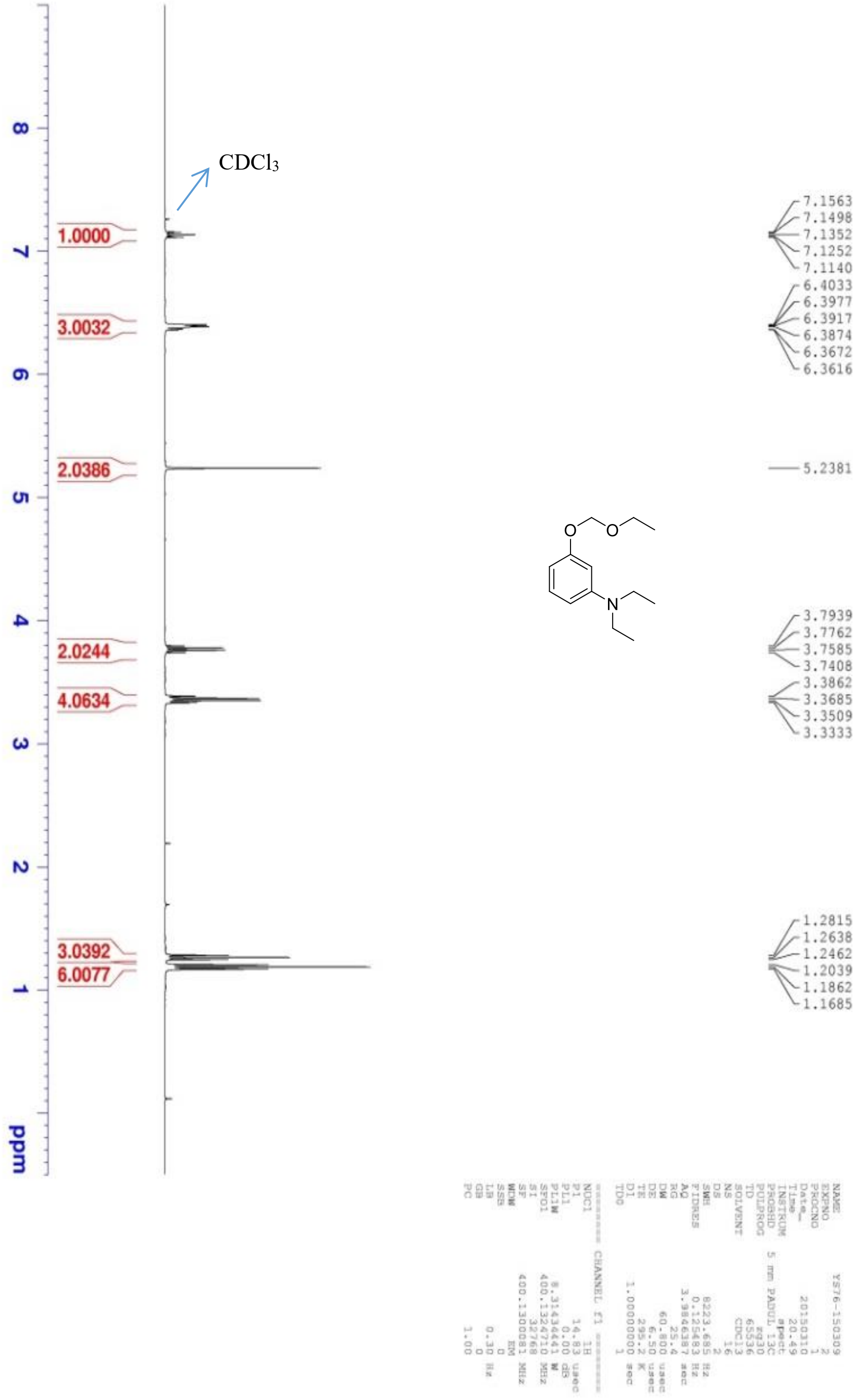

Figure S1. ^{1}H NMR spectrum of compound **2**

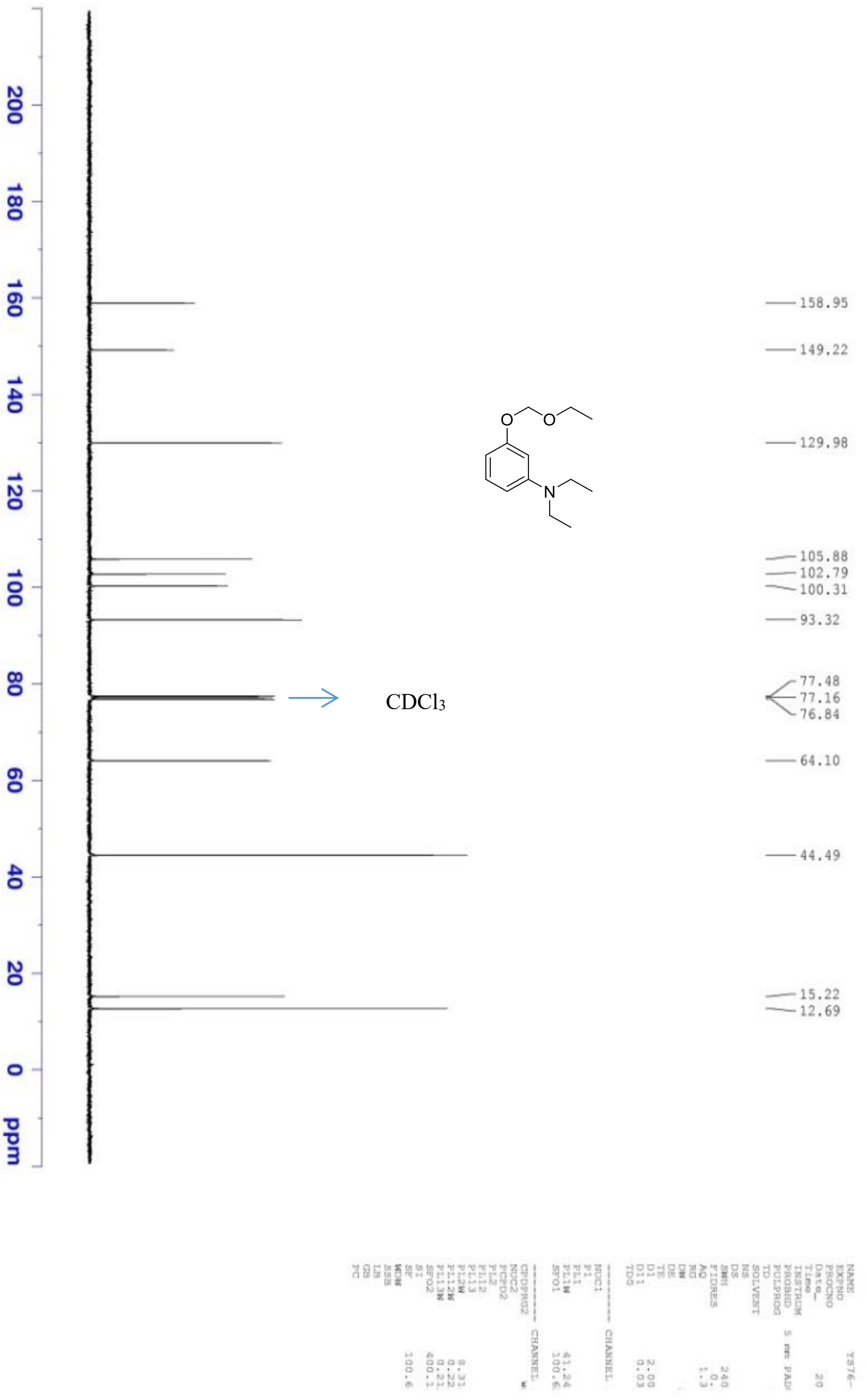

Figure S2. ^{13}C NMR spectrum of compound **2**

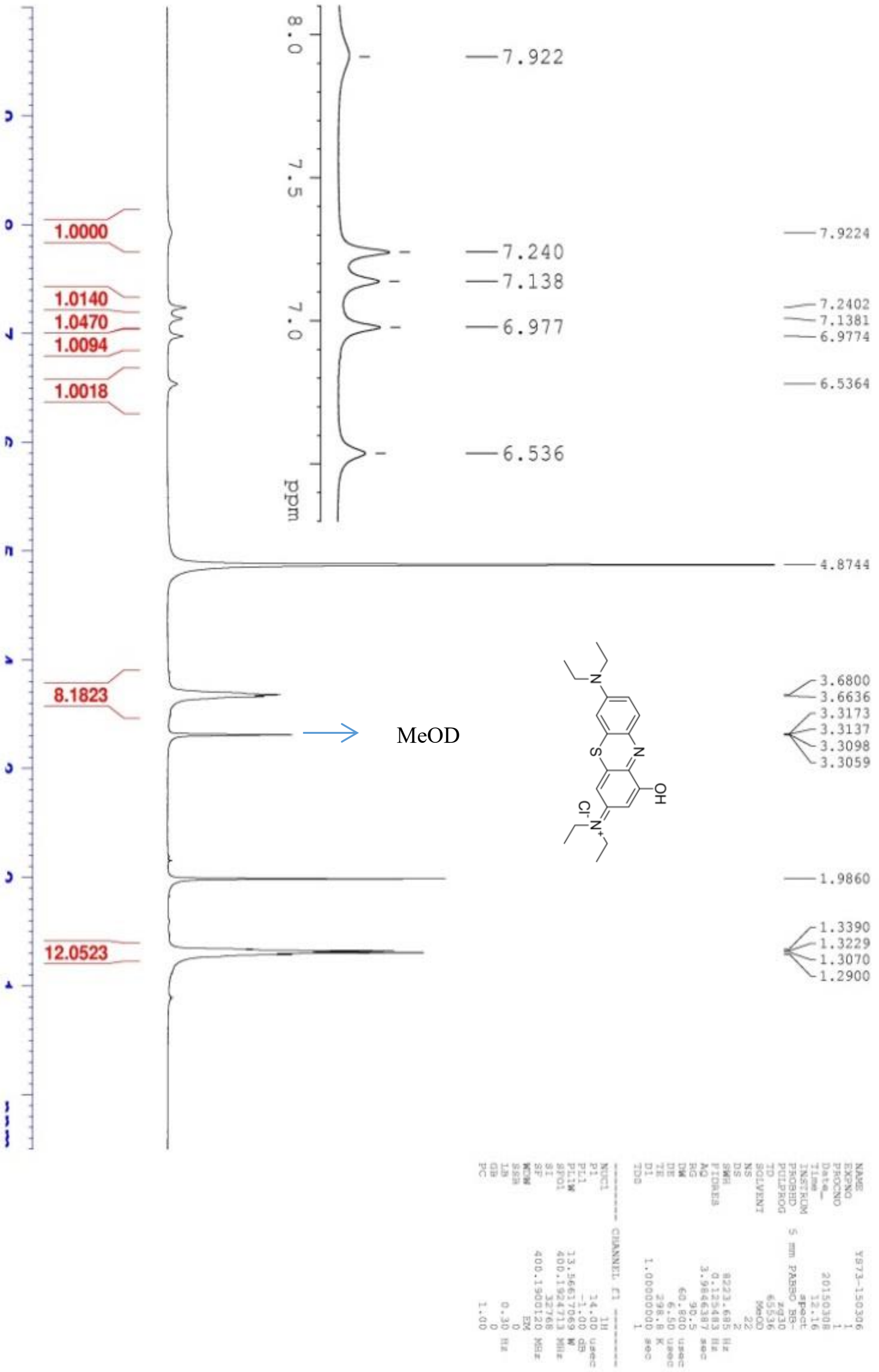

Figure S3. ^{1}H NMR spectrum of compound **5**

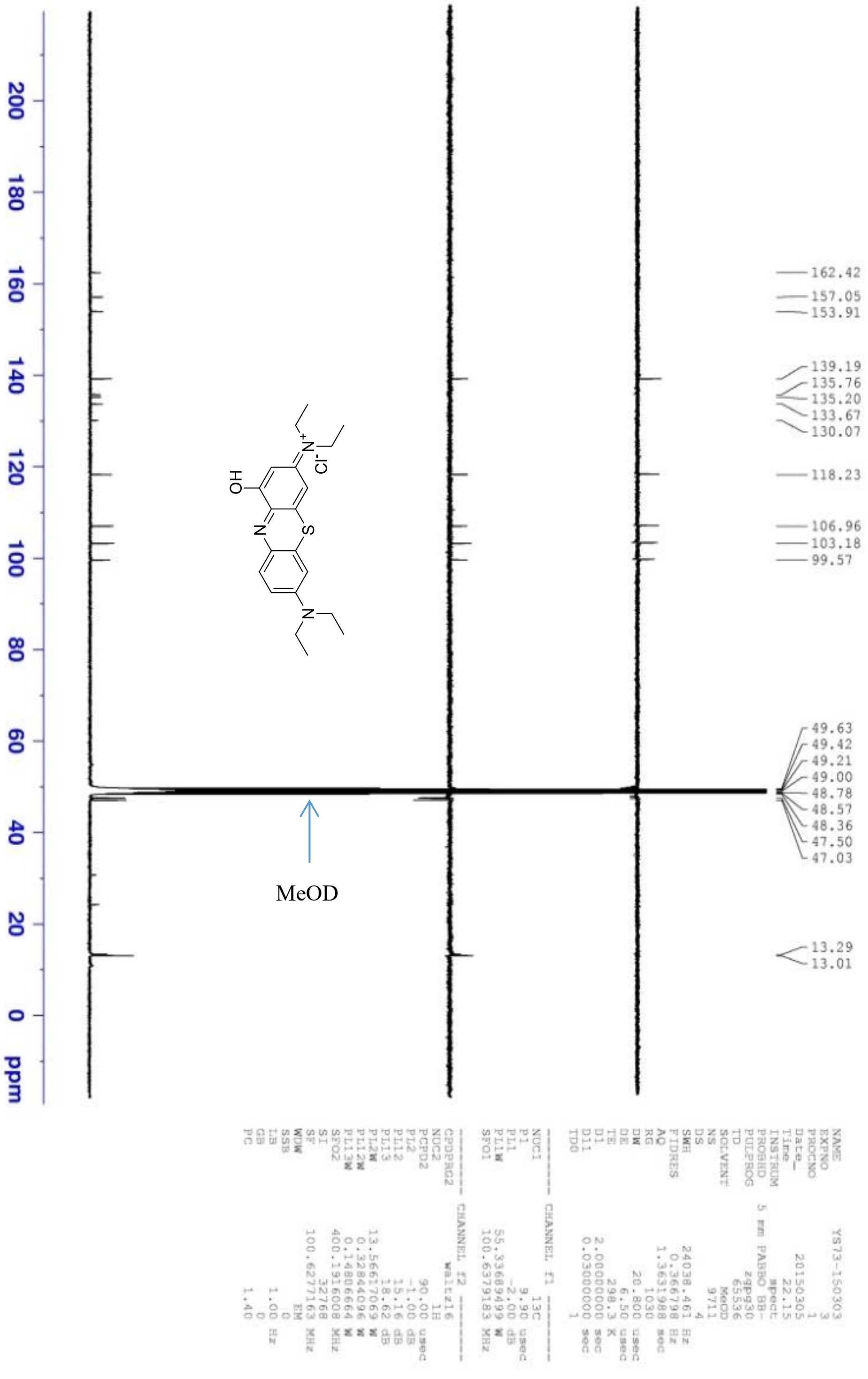

Figure S4. ^{13}C NMR spectrum of compound **5**

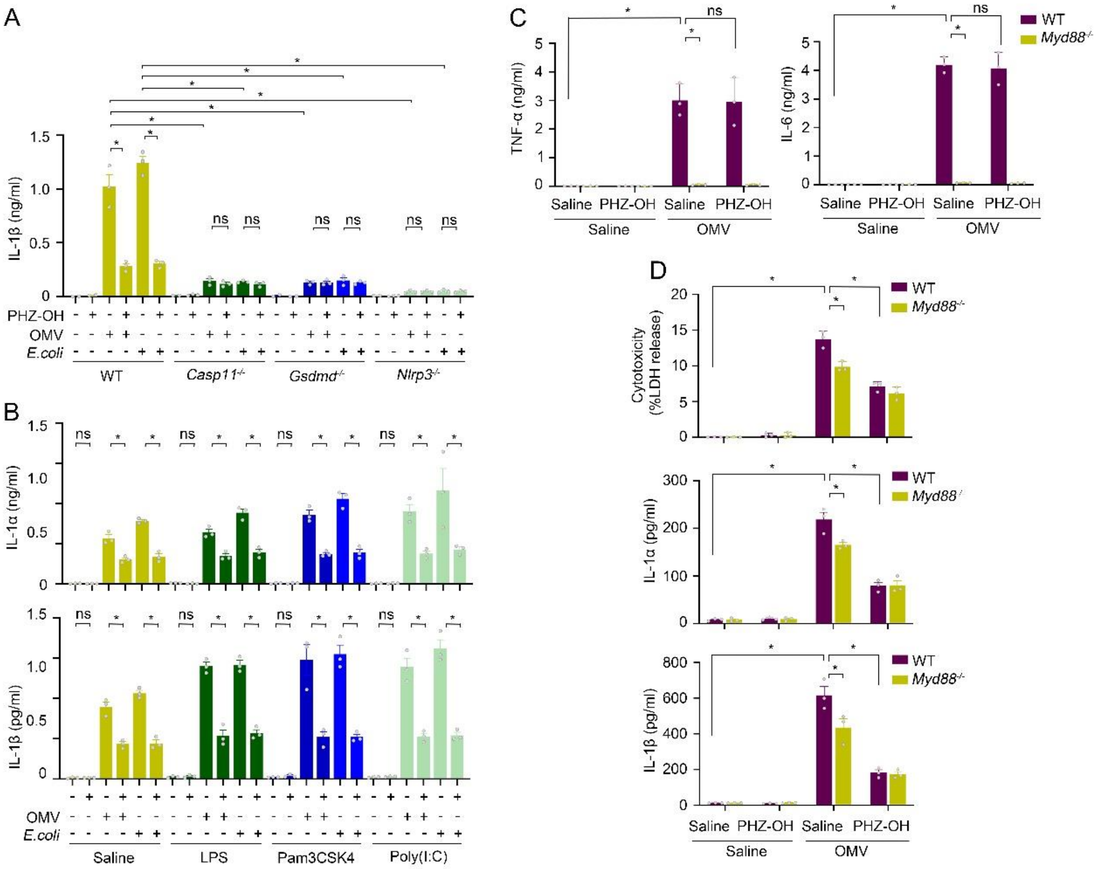

Figure S5. Cytotoxicity and cytokine secretion of macrophages under various treatments. (A) IL-1β release of WT, *Casp11*$^{-/-}$, *Gsdmd*$^{-/-}$, or *Nlrp3*$^{-/-}$ mice peritoneal macrophages under a treatment of PHZ-OH (5 μM), and/or a challenge of OMV (10 μg/mL) or *E.coli* (MOI of 10) for 16 h; (B) IL-1α/β secretion of macrophages primed using LPS (1 μg/mL), Pam3cSK4 (1 μg/mL) or Poly(I:C) (1 μg/mL) for 3 h and challenged with OMV (10 μg/mL) or *E.coli* (a MOI of 10) for 16 h in the presence or absence of PHZ-OH (5 μM); (C and D) WT or *Myd88*$^{-/-}$ macrophages were treated with saline or PHZ-OH (5 μM) and challenged with OMV (10 μg/mL) for 16 h or not. (C) TNFα and IL-6 secretion; (D) Cytotoxicity and IL-1α/β. Data are shown as mean ± SEM of three independent experiments. * *P*<0.05.

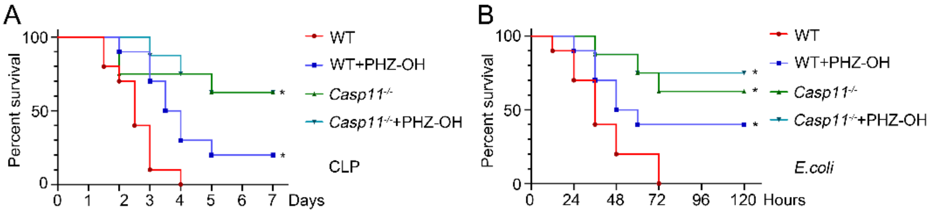

Figure S6. Survival rate of mice under a challenge of CLP or *E. coli.* (A) Survival rate of WT or *Casp11*$^{-/-}$ mice treated with or without PHZ-OH (5 mg/kg) 30 min after a challenge of CLP; (B) Survival rate of WT or *Casp11*$^{-/-}$ mice treated with or without PHZ-OH (5 mg/kg) 30 min after a challenge of *E. coli* (10^9 CFU per mouse). * $P<0.05$.

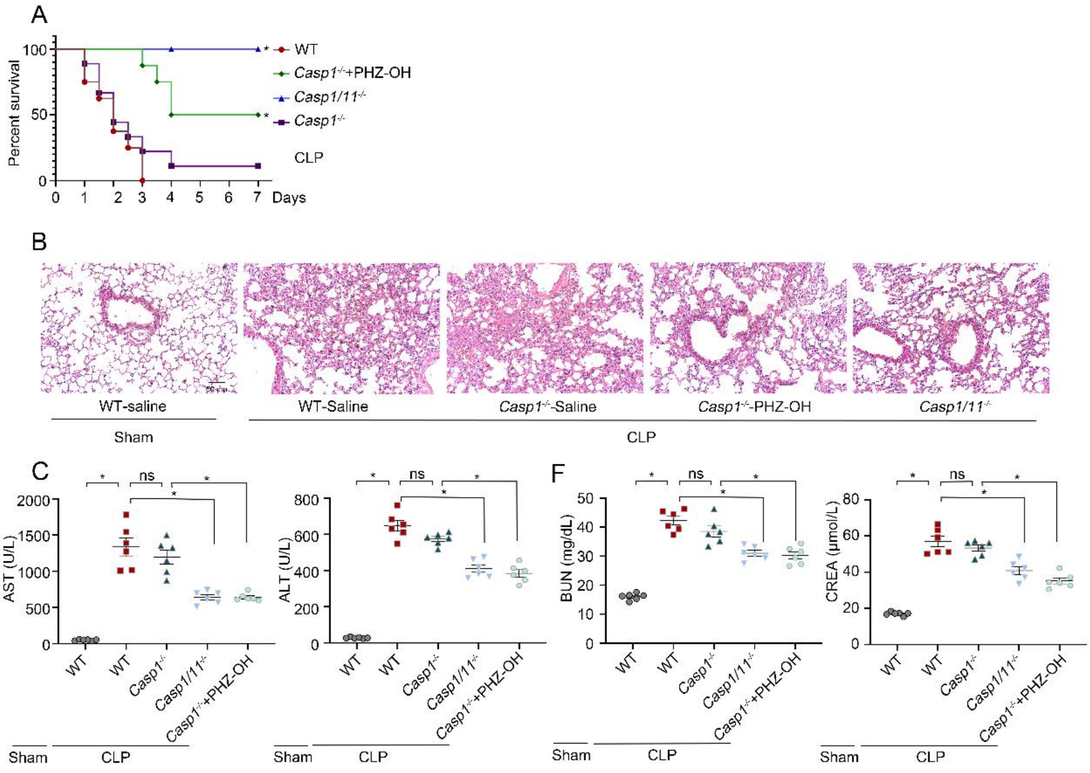

Figure S7. Survival rates and organ injury of WT or gene-modified mice challenged with CLP. (A) Survival rates of WT, *Casp1*$^{-/-}$ and *Casp1/11*$^{-/-}$ mice treated with PHZ-OH (5 mg/kg) or not 30 min prior to a challenge of CLP; (B-D) Lung injury (D), liver (C) and kidney dysfunction (D) of WT, *Casp1*$^{-/-}$ and *Casp1/11*$^{-/-}$ treated with PHZ-OH (5 mg/kg) 30 min prior to a challenge of CLP, using shammed mice as the negative control. Data are mean ± SEM of six mice in one experiment. * *P*<0.05. ns, not significant. Scale bar = 50 μm.

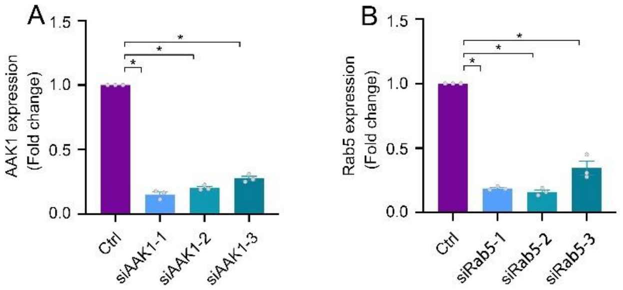

Figure S8. Knockdown effects of AAK1 and Rab5. (A) AAK1 expression level of macrophages treated with AAK1 siRNA for 48 h. (B) Rab5 expression level of macrophages treated with Rab5 siRNA for 48 h. Data are shown as mean ± SEM of three independent experiments. * *P*<0.05.

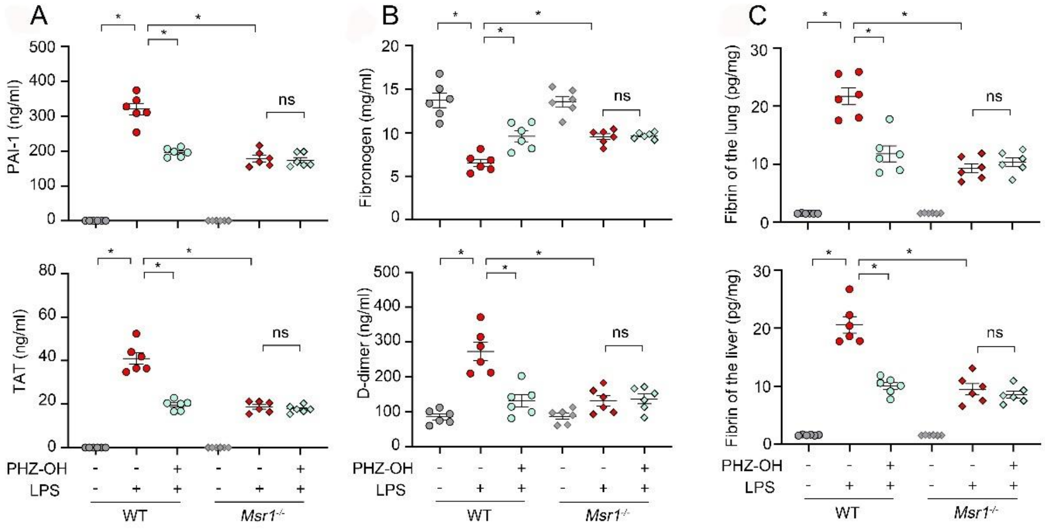

Figure S9. Coagulation of WT and *Msr1*-deficient mice challenged with LPS. (A-C) Plasma concentrations of PAI-1 and TAT (A), fibrinogen and D-dimer (B), and Fibrin concentrations of the lung and the liver in saline-treated or LPS-challenged (0.4 mg/kg LPS for 7h and a following 10 mg/kg LPS for 12h) WT and *Msr1*$^{-/-}$ mice under an intervention of PHZ-OH (5 mg/kg) or not. Data are mean ± SEM of six mice in one experiment. * *P*<0.05. ns, not significant.

Table S2. Binding energy of PHZ-OH with candidate proteins using molecular docking

Protein	PDB ID	NP			CGB			BN
		X	Y	Z	X	Y	Z	
AAK1	4WSQ	60	60	60	-4.287	-15.223	-76.878	-8.7
GAK	4C58	60	60	68	30.006	19.736	-50.13	-8
MD-1	3B2D	60	60	60	32.48	-28.756	-12.37	-7.8
INTEGRIN αII	1M1X	126	126	84	8.96	45.838	31.289	-7.6
MD-2	2E59	60	60	60	-6.205	19.791	13.202	-7.3
EGFR	5D41	60	60	60	-30.229	28.137	20.857	-7.1
INTEGRIN α V	3FCS	126	80	118	-83.844	-65.002	-87.105	-7.1
INTEGRIN β1	4WK2	100	88	56	16.859	15.159	-30.528	-7
CXCR4	3OE8	90	96	70	-49.203	42.515	20.171	-7
PIP5K	6CMW	60	60	60	-20.17	12.453	-17.267	-6.7
AP2	2XA7	60	60	60	56.342	-43.595	-7.71	-6.3
SNX9	2RAK	98	104	60	11.695	35.679	21.044	-6.2
PI3K	3APD	66	62	66	49.364	18.025	29.674	-6.2
L-selectin	5VC1	60	90	90	-4.319	36.635	39.877	-6
INTEGRIN β3	1M1X	104	104	92	17.766	28.798	34.86	-6
RP105	3B2D	78	80	66	38.045	-10.952	-10.917	-6
SR-A6	2OY3	60	60	60	5.534	-2.053	17.433	-5.97
TLR9	3WPF	92	96	76	22.69	17.36	30.605	-5.86
Dynamin-1	3ZVR	84	84	102	-101.249	31.093	-101.455	-5.8
E-selectin	4C16	88	82	92	38.404	18.281	4.193	-5.7
INTEGRIN β2	4NEH	118	106	104	-49.375	38.635	63.056	-5.7
SR-A4	2OX9	60	60	60	77.764	75.829	49.905	-5.6
HSP70	6S02	60	60	60	34.091	79.044	58.682	-5.5
P-selectin	1G1Q	88	88	60	64.333	-2.417	16.694	-5.5
OCRL	4CMN	96	94	104	-17.849	28.5	-18.827	-5.4
CD14	4GLP	94	60	90	47.926	57.119	0.766	-5.4
TLR4	2Z63	78	86	122	19.487	-36.555	4.372	-5.3
SR-A1	6J02	60	40	40	16.009	-18.603	-3.57	-5.1
SR-B2	5LGD	60	60	60	-40.505	-34.565	52.944	-5.1
SR-B3	4TW2	60	60	60	14.856	40.351	-0.512	-5.1
TREM-1	1SMO	60	60	60	17.683	42.527	3.784	-5.1
Intersectin-1	3JV3	60	60	60	-29.949	13.656	12.614	-4.9
Clathrin	5M5R	40	40	40	-8.301	-25.356	15.786	-4.5
SR-I1	5JFB	60	60	60	15.793	8.693	-5.516	-4.4
FCHO1	5JP2	60	60	60	-28.914	14.653	-15.752	-4.4
Moesin	1EF1	60	60	60	12.053	123.958	59.384	-4.4

AAK1: AP2-associated protein kinase 1; BN: binding energy (kcal/mol); CGB: center grid box; EGFR: Epidermal growth factor receptor; FCHO1: FR: flexible residues; GAK: Cyclin-G-associated kinase; HSP70: Heat shock protein 70; NECAP2: Adaptin ear-binding coat-associated protein 2; NP: number of points; OCRL: PIP5K: Phosphatidylinositol-4-phosphate 5-kinase; PI3K: Phosphatidylinositol-4,5-bisphosphate 3-kinase; Ref: references; SNX9:Sorting nexin-9; TLR: Toll-like receptor.
