# Supplementary material for: Novel 1-hydroxy phenothiazinium-based derivative protects against bacterial sepsis by inhibiting AAK1-mediated LPS internalization and caspase-11 signaling
Source: Cell Death Dis. 2022 Aug 18;13(8):722. doi: 10.1038/s41419-022-05151-7 (PMC9387894; doi:10.1038/s41419-022-05151-7)
Supplement: Supplementary file 2 — Uncropped WB images [file 41419_2022_5151_MOESM2_ESM.pdf]

Figure 1F IL-1α

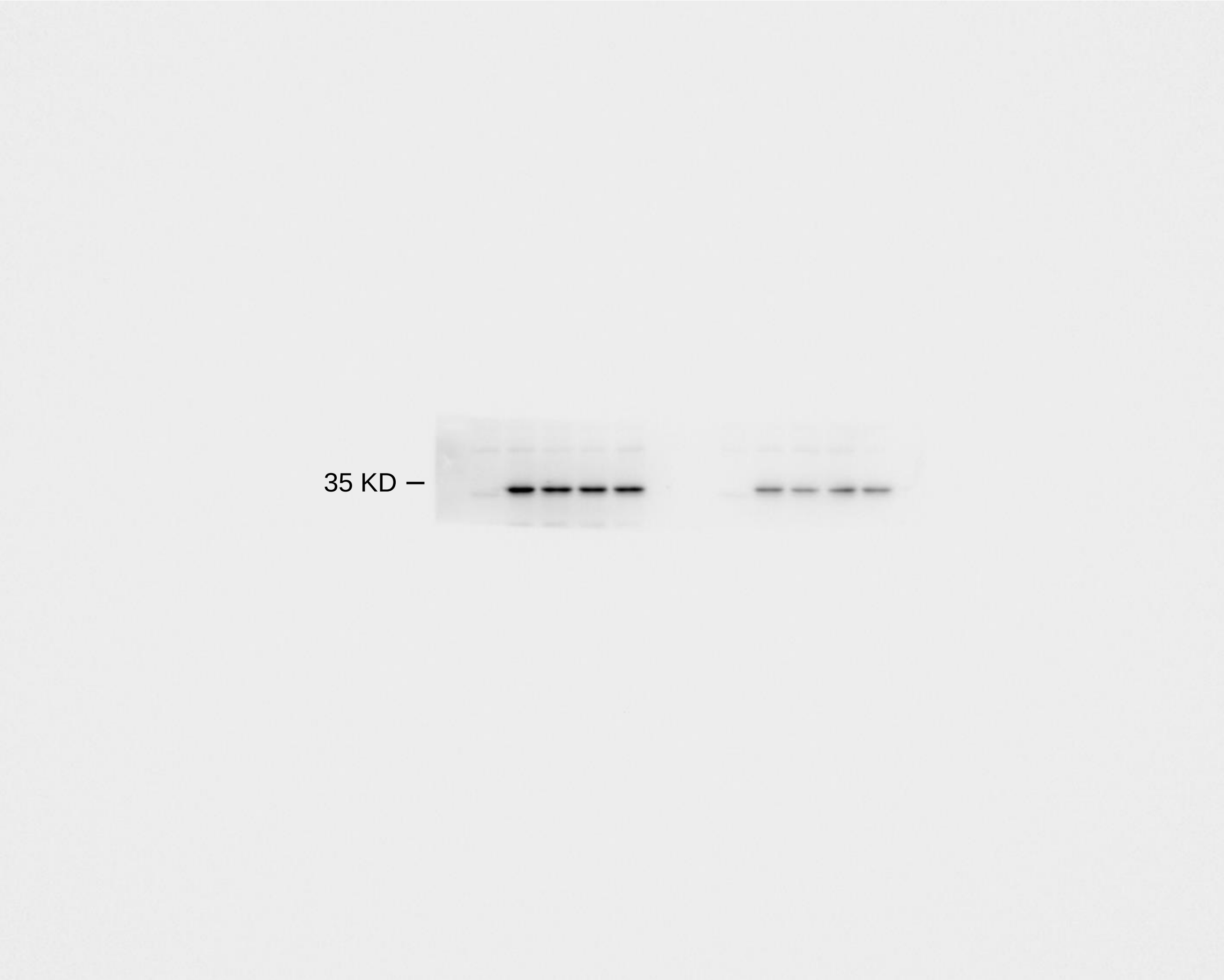

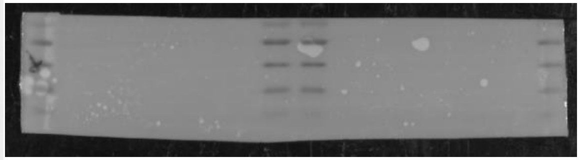

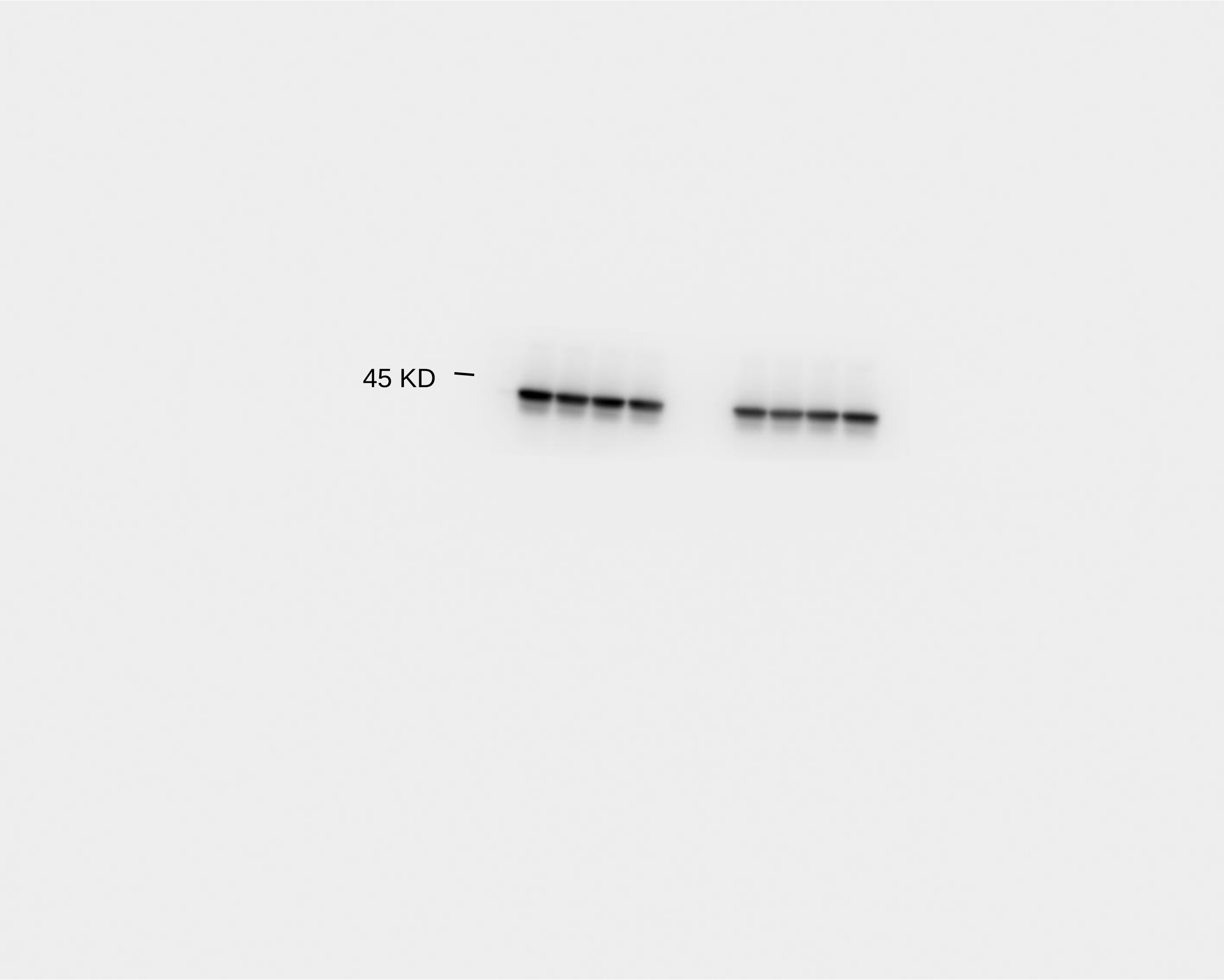

Figure 1F IL-1β

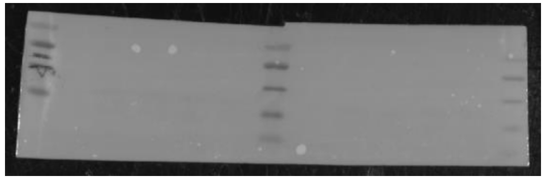

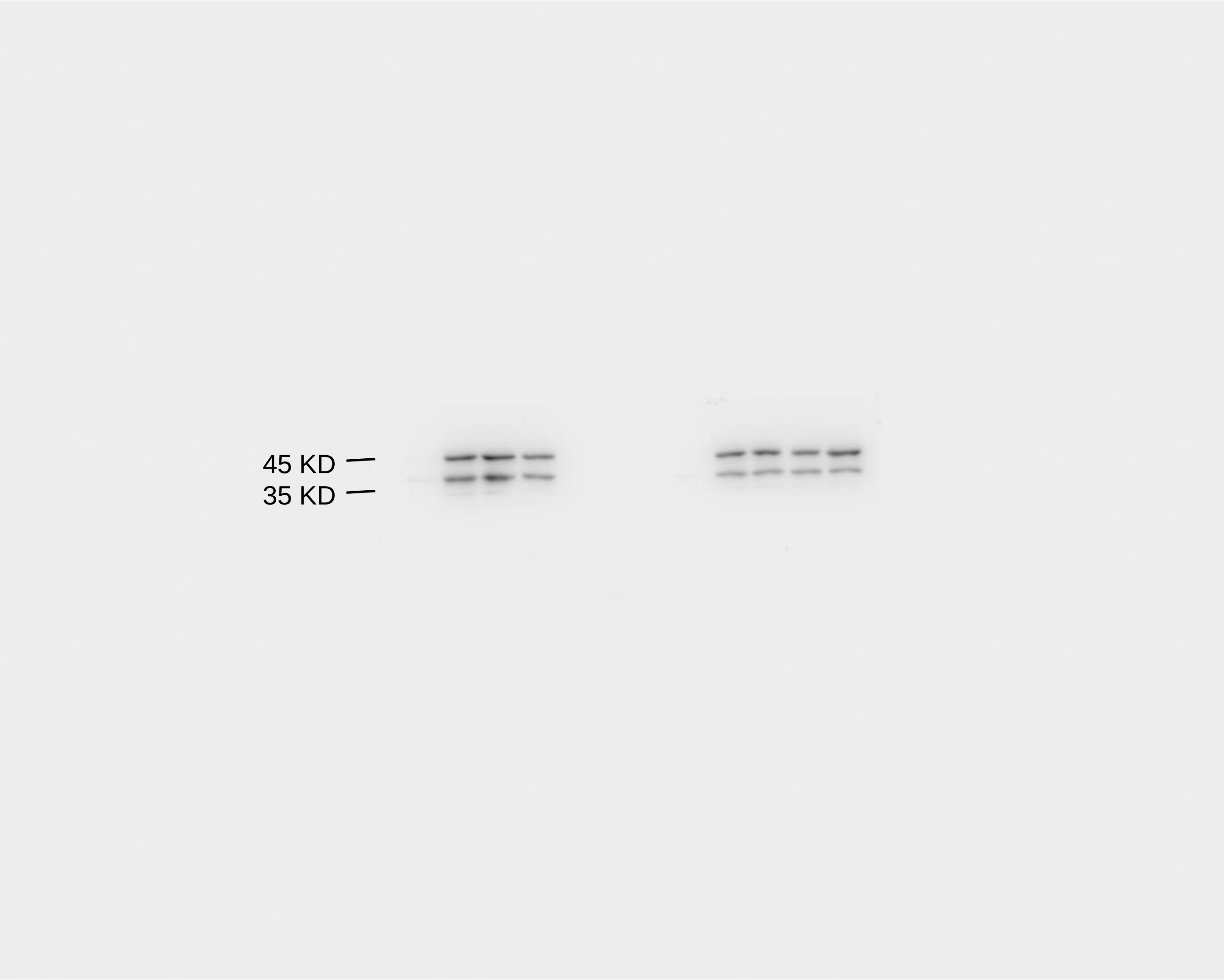

Figure 1F Caspase-11

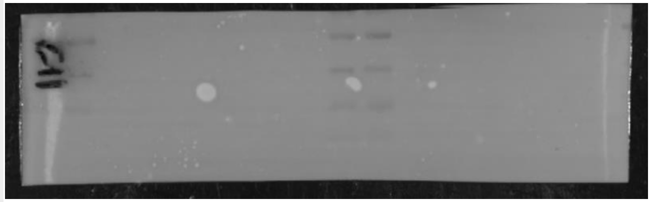

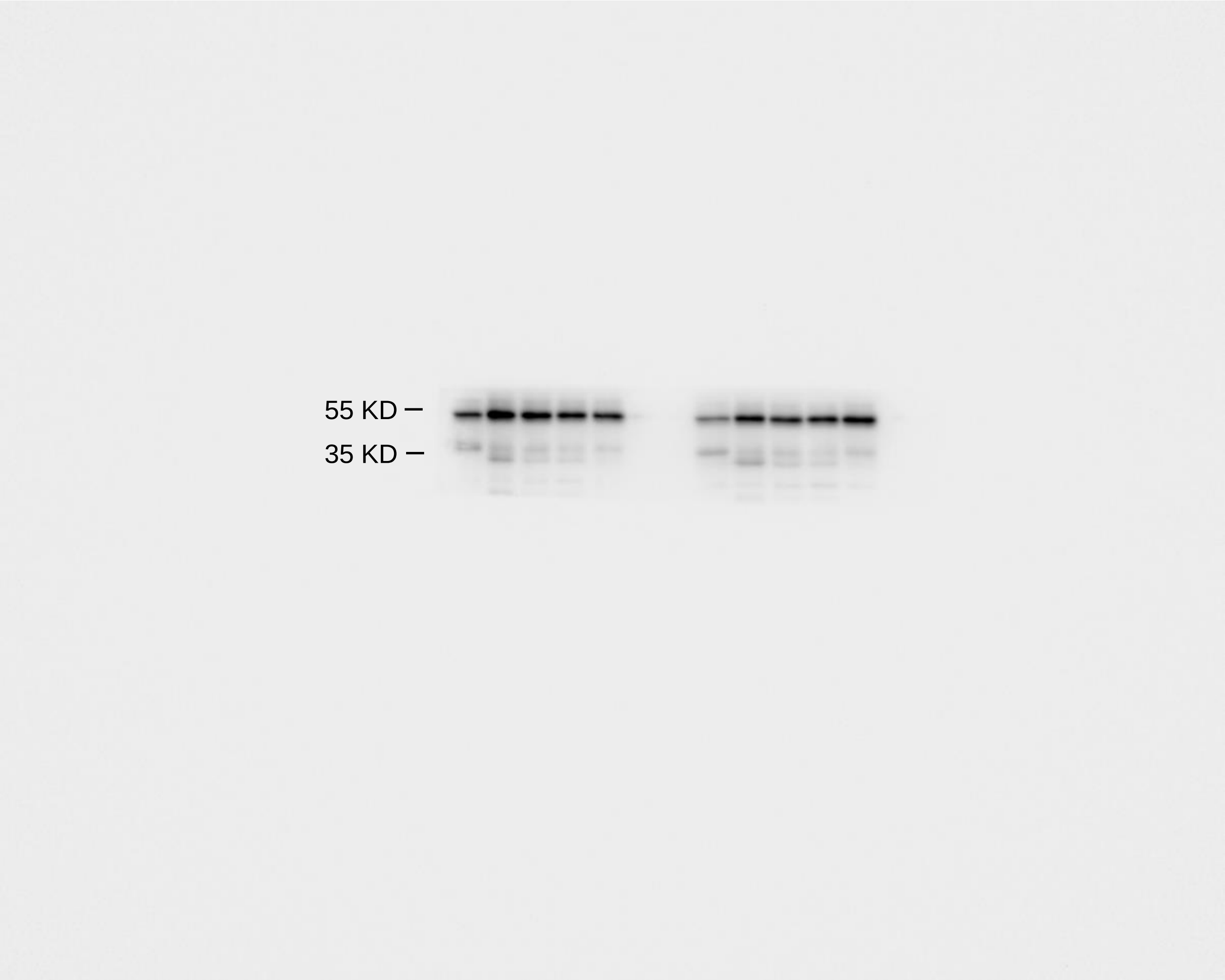

Figure 1F GSDMD

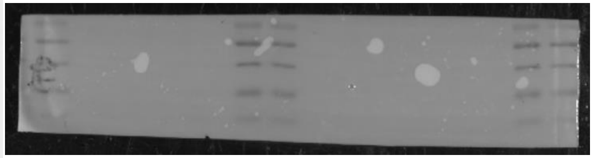

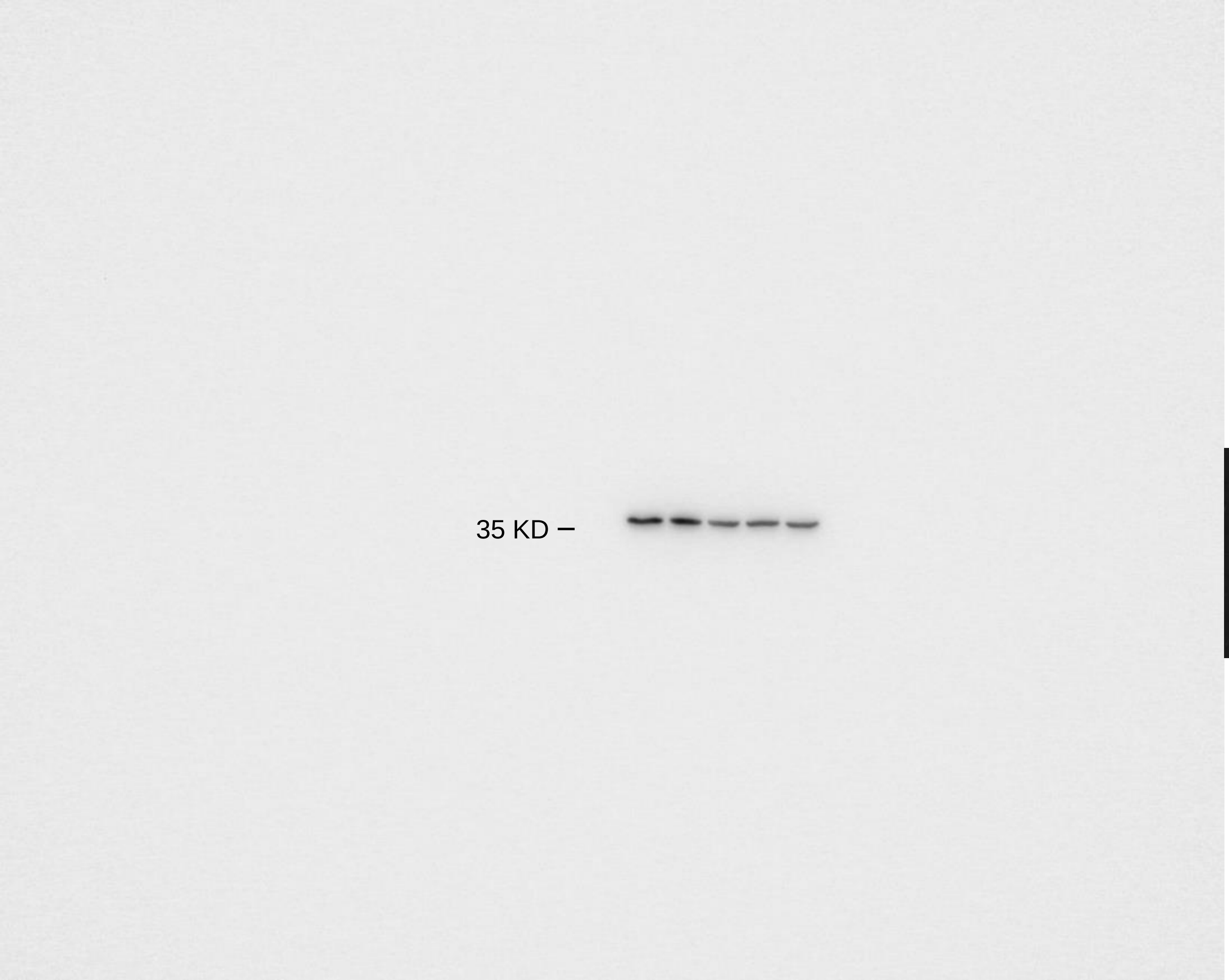

Figure 1F β-actin

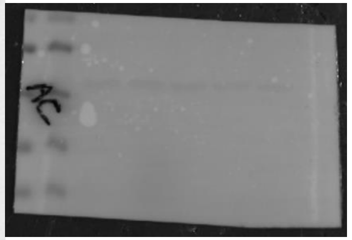

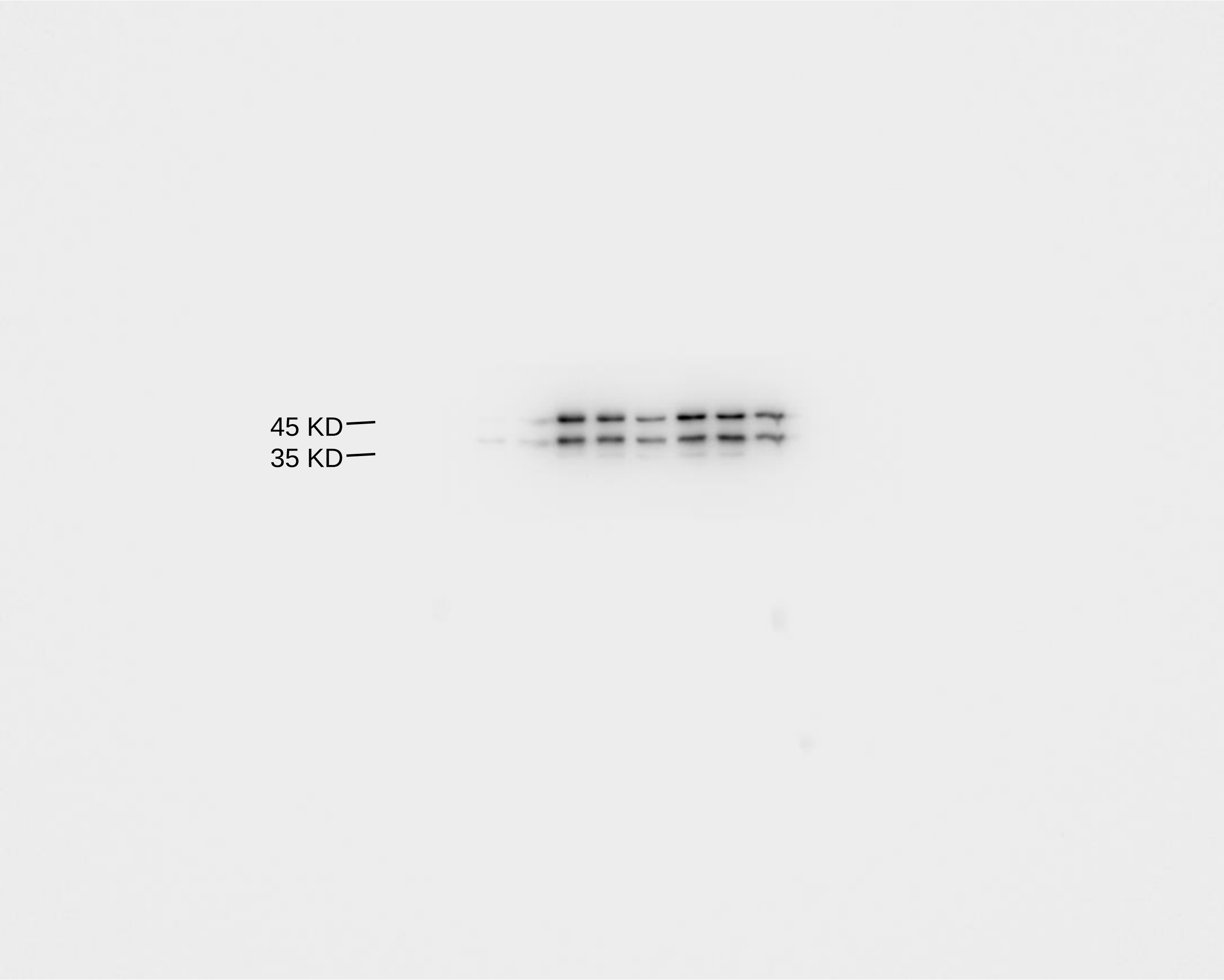

Figure 2H Caspase-11

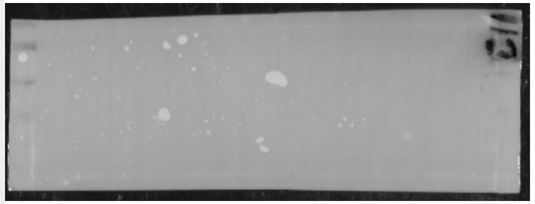

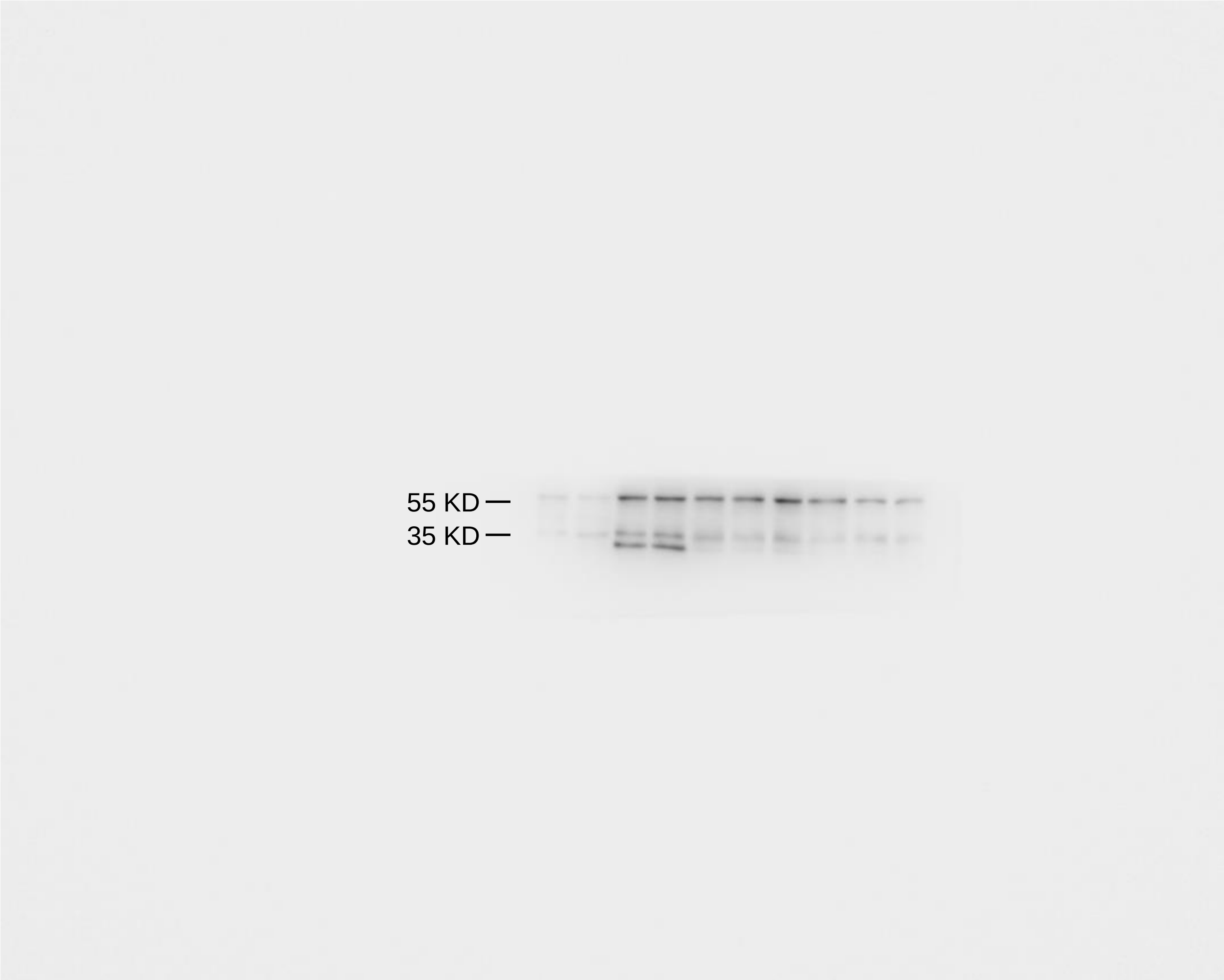

Figure 2H GSDMD

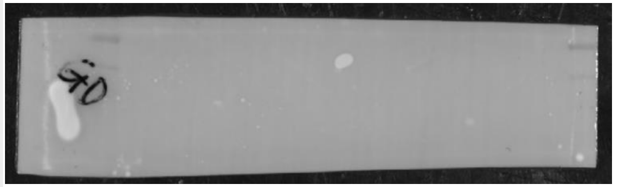

Figure 2H β-actin

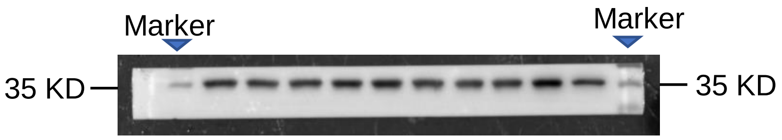

The merged image with whole PVDF membrane is shown above. The original image includes unpublished data of another study and is thus not displayed here.
